# Exosomal Long Non-Coding RNAs in Lung Diseases

**DOI:** 10.3390/ijms21103580

**Published:** 2020-05-19

**Authors:** Christophe Poulet, Makon-Sébastien Njock, Catherine Moermans, Edouard Louis, Renaud Louis, Michel Malaise, Julien Guiot

**Affiliations:** 1 Department of Rheumatology, University Hospital of Liège (CHULiege), 4000 Liège, Belgium; ms.njock@chuliege.be (M.-S.N.); michel.malaise@chuliege.be (M.M.); 2 Fibropôle Research Group, University Hospital of Liège (CHULiege), 4000 Liège, Belgium; edouard.louis@uliege.be (E.L.); r.louis@chuliege.be (R.L.); 3 GIGA-I3 Research Group, GIGA Institute, University of Liège (ULiege) and University Hospital of Liège (CHULiege), 4000 Liège, Belgium; c.moermans@chuliege.be; 4 Department of Respiratory Diseases, University Hospital of Liège (CHULiege), 4000 Liège, Belgium; 5 Department of Gastroenterology, University Hospital of Liège (CHULiege), 4000 Liège, Belgium

**Keywords:** lncRNA, H19, MEG3, MALAT1, HOTAIR, exosome, COPD, asthma, IPF, lung cancer

## Abstract

Within the non-coding genome landscape, long non-coding RNAs (lncRNAs) and their secretion within exosomes are a window that could further explain the regulation, the sustaining, and the spread of lung diseases. We present here a compilation of the current knowledge on lncRNAs commonly found in Chronic Obstructive Pulmonary Disease (COPD), asthma, Idiopathic Pulmonary Fibrosis (IPF), or lung cancers. We built interaction networks describing the mechanisms of action for COPD, asthma, and IPF, as well as private networks for H19, MALAT1, MEG3, FENDRR, CDKN2B-AS1, TUG1, HOTAIR, and GAS5 lncRNAs in lung cancers. We identified five signaling pathways targeted by these eight lncRNAs over the lung diseases mentioned above. These lncRNAs were involved in ten treatment resistances in lung cancers, with HOTAIR being itself described in seven resistances. Besides, five of them were previously described as promising biomarkers for the diagnosis and prognosis of asthma, COPD, and lung cancers. Additionally, we describe the exosomal-based studies on H19, MALAT1, HOTAIR, GAS5, UCA1, lnc-MMP2-2, GAPLINC, TBILA, AGAP2-AS1, and SOX2-OT. This review concludes on the need for additional studies describing the lncRNA mechanisms of action and confirming their potential as biomarkers, as well as their involvement in resistance to treatment, especially in non-cancerous lung diseases.

## 1. Introduction

The purpose of this review is to summarize the current knowledge in the field of long non-coding RNAs (lncRNAs) and exosomal-lncRNAs involved in lung diseases such as Idiopathic Pulmonary Fibrosis (IPF), Chronic Obstructive Pulmonary Disease (COPD), asthma, and lung cancer. To this aim, we collected information from RNAseq and microarray data when available. Furthermore, we standardized each gene and microRNA (miRNA) name to the official gene symbols and miRNA nomenclatures.

The following three significant steps describe the overall methodology used to process the literature. First, using the PubMed database, we seek for publications related to lncRNAs in each disease. Secondly, we standardized the gene names using the official gene symbols and the Ensembl identifier. Finally, we overlapped the standardized gene lists from each disease and identified ten lncRNAs associated with at least two of the diseases mentioned above. These ten lncRNAs are H19, MALAT1, MEG3, FENDRR, CDKN2B-AS1, TUG1, HOTAIR, GAS5, LINC00861, and CCDC18-AS1.

After a brief introduction to the diseases mentioned above, we will cover the selected lncRNAs one by one. We will describe their known mechanisms of action, their potential as biomarkers, as well as their involvement in treatment resistances. We will then compile the three steps into interaction networks and tables. Finally, we will report the current knowledge about exosomal lncRNAs in lung cancers. Additionally, we listed all the abbreviations and gene definitions in the abbreviation section with hyperlinks redirecting to Ensembl, NONCODE, or HUGO nomenclature databases for the lncRNAs, the miRBase database for the miRNAs, and the KEGG database for the pathways.

### 1.1. Overview of Lung Diseases Covered in the Current Review

#### 1.1.1. Idiopathic Pulmonary Fibrosis (IPF)

Idiopathic pulmonary fibrosis is a progressive fibrosing lung disease of unknown aetiology. It is associated with a high morbidity/mortality rate of 3–5 years without specific anti-fibrotic therapy [1,2,3,4]. The prevalence of IPF in Europe ranged from 1.25 to 23.4 cases per 100,000 while being between 14 and 27.9 cases per 100,000 in the USA. The prevalence appears to increase along with age and to be higher among males than females [5].

Despite new anti-fibrotic therapies reducing its evolution, IPF is still challenging to manage. The main particularity of IPF is its sole lung involvement with typical fibroblastic activation. Nevertheless, the physiopathology is still not well known and needs further translational studies. Additionally, the diagnostic approach remains challenging and requires a multidisciplinary approach, including respiratory specialists, radiologists, pathologists, and thoracic surgeons. Besides, the radiological images can be nonspecific, which often requires a lung biopsy to produce an accurate diagnosis. Subsequently, clinicians are urging for specific biomarkers to diagnose the IPF at an early stage to identify patients with a high risk of a rapid flare-up [6,7].

#### 1.1.2. Chronic Obstructive Pulmonary Disease (COPD)

COPD is a chronic inflammatory obstructive lung disease, which will probably become the third leading cause of death in 2030, according to the World Health Organization (WHO). It is linked to smoking habits and is characterized by an airflow limitation, which interferes with normal breathing and reduces the potential of physical activity. In 2010, the scientific community estimated the prevalence of COPD at 384 million cases, with a global incidence of 11.7% at a 95% confidence interval (CI) 8.4–15.0% [8].

The clinicians establish the diagnosis of COPD with the spirometry. This simple test measures how deeply a person can breathe and how fast air can move into and out of the lungs. COPD symptoms associate chronic cough, sputum production, dyspnea, and a history of exposure to risk factors for the disease. The current treatment strategy uses bronchodilators and inhaled corticosteroids in case of a frequent exacerbation. However, for the most severe cases, it remains challenging to reduce the acute exacerbations and airflow limitation, leading to a significant decrease in the patient’s quality of life. New biomarkers are, therefore, required to detect patients with a high risk of frequent exacerbations, develop specific targeted therapies (GOLD guidelines), and improve the global outcome of COPD patients [9].

#### 1.1.3. Asthma

Asthma is not a single disease, but a complex chronic inflammatory disease of the airways. Indeed, several subtypes of bronchial asthma, also called phenotypes, have different therapeutic and prognostic implications. According to the Global Initiative for Asthma (GINA), it affects all age groups of about 5% to 10% of the world population. Asthma is specifically characterized by a transient obstruction of the respiratory tract, secondary to bronchoconstriction, or bronchial inflammation. Inflammatory phenotypes can now classify asthmatic patients and allow personalized therapies, thus urging for the identification of biomarkers linked to the widely prevalent disease [10].

#### 1.1.4. Lung Cancer

Lung cancer is still the leading cause of cancer-related death in the world despite new therapeutic progress like checkpoint inhibitors. Its prevalence varies worldwide due to tobacco habits, air quality, race/ethnicity, gender, age, and education/income [11]. In 2018, the WHO reported 2.09 million cases of lung cancers around the world. The two main categories of lung cancer are small-cell lung carcinoma (SCLC) and non-small cell lung carcinoma (NSCLC). According to cancer.org, about 13% of all lung cancers are SCLC, and 84% are NSCLC. The overall survival rate at five years is still <20% despite new specific anti-tumoral therapies as checkpoint inhibitors. Precisely, the survival rate for the extensive stage of SCLC reaches seven months, whereas it is up to 11 months for the widespread disease of NSCLC. These poor survival rates are mainly due to late-stage diagnosis [12].

Subsequently, a new comprehensive approach of the molecular mechanisms is crucial to identify new therapeutic targets for the patients. Finding new biomarkers is, therefore, one of the primary objectives to detect lung cancer at the earliest stages. Lung cancer-derived exosomes are known to represent the cell of origin in many aspects. They need to be intensively studied to better understand cell-cell communication and cancer proliferation.

### 1.2. Exosomes

Exosomes are nanovesicles of about 30–150 nm, which are generated within late endosomes/multivesicular bodies (MVBs). They are released to the extracellular microenvironment through MVB fusion to the plasma membrane and exocytosis [13,14]. These vesicles contain many components of the parental cell, including cell-surface proteins, lipids, metabolites, and genetic material (DNA, mRNA, non-coding RNAs including miRNAs and lncRNA) [15,16]. The secretion of exosomes into biological fluids (e.g., bronchoalveolar lavage fluid, saliva, sputum, plasma) confer them promising diagnostic/prognostic value. In a recent study, the characterization of miRNAs content of exosomes from the sputum of patients with IPF enabled to identify a unique signature of 3 altered miRNAs hsa-miR-142-3p, hsa-miR-33a-5p, and hsa-let-7d-5p [17]. Furthermore, the study of the functional properties of exosomes in the context of lung diseases could open new avenues of therapeutic approaches [18,19]. Depending on their composition, exosomes can maintain cellular homeostasis [20], or alter the functional properties of the recipient cell and impact the progression of the disease [21,22,23,24]. Microenvironment plays an important role in the composition of exosomes. For example, the exposure of lung microenvironment to noxious stimuli (e.g., cigarette smoke (CS), allergens, infections, air pollutants) induces the release of airway exosomes enriched with pro-inflammatory/pro-fibrotic components which participate in the progression of lung diseases [21,22,23,24]. Importantly, with the new RNA technologies combined with the recent efforts to define stable reference genes, we are now able to identify better the differentially expressed exosomal-lncRNAs that are involved in lung diseases [25,26,27].

#### 1.2.1. Exosomes Are Essential Actors of Intercellular Communication

**Figure 1:**
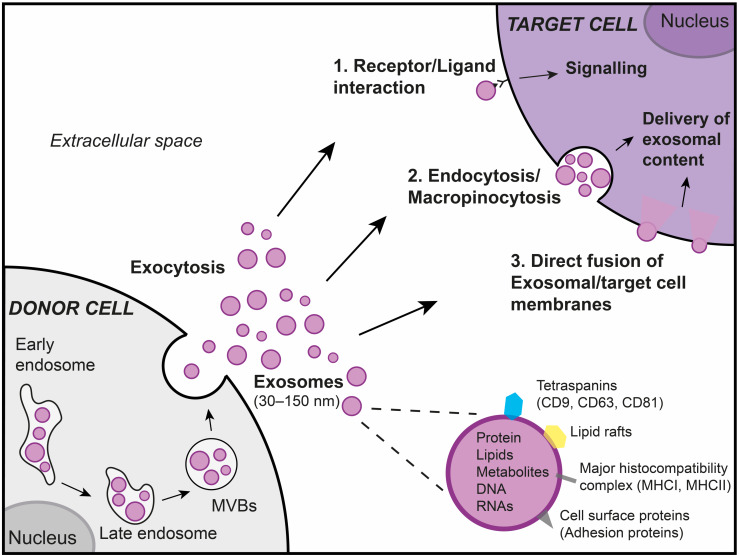
Exosomes in intercellular communication. Exosomes are nanovesicles (30–150 nm), which originate from the endosomal pathway by the formation of the early endosomes, late endosomes, and, ultimately, multivesicular bodies (MVBs). These vesicles are released to the extracellular microenvironment through MVB fusion to the plasma membrane and exocytosis. Exosomes contain many components of donor cells, including cell-surface proteins, lipids, metabolites, and genetic material, which confer them functional properties. Exosomes can transfer information to the target cell by (1) interacting with the cell surface, via a receptor-mediated mechanism, or by delivering its content to the target cell through (2) endocytosis, macropinocytosis or (3) through a direct fusion of exosomal membrane with the plasma membrane.

By the presence of specific surface components as well as the packaging of proteins, lipids, and genetic material, exosomes are bio-effector units that can regulate the properties of target cells. Distinct mechanisms associated with exosome uptake are involved in intercellular communication [28,29,30]. As described in Figure 1, exosomes can transfer the information to target cells via a receptor-mediated mechanism without the delivery of their content. For instance, the high level of Intercellular Adhesion Molecule 1 on exosomes, from mature dendritic cells (DCs), is critical for efficient naive T-cell priming [31]. Furthermore, DC-derived exosomes can carry functional major histocompatibility complex class I and class II molecules that can be loaded with specific peptides to activate viral-specific peripheral CD8+ T cells [31,32]. The other mechanisms involved in intercellular communication are the delivery of exosomal cargo to target cells through macropinocytosis [33,34,35], clathrin-dependent endocytosis [33], or membrane fusion [36].

#### 1.2.2. Exosomes Are Playing an Essential Role in Lung Diseases

In a physiological context, various cell types from the lung microenvironment participate in the regulation of lung homeostasis via the secretion of exosomes presenting anti-inflammatory/anti-fibrotic properties [20,37,38,39]. Noxious stimuli exposure (e.g., CS, allergens, infections, air pollutants) can impact nucleic acid cargo (miRNAs, lncRNAs) of lung-derived exosomes and alter their protective properties. Indeed, CS exposure induces the alteration of the composition of bronchial epithelial-derived exosomes composition, with an upregulation of hsa-miR-21 and hsa-miR-210 [22,23], which in turn dysregulates several cellular processes associated with the progression of the COPD. In the asthma context, several studies have reported an alteration of inflammatory-related exosomal miRNAs from airway biofluids (BALF, and sputum supernatants) [21,24]. In conclusion, lung exosomes released in the pathological context present an alteration of their composition, which in turn may impact the progression of lung diseases.

### 1.3. Long Non-Coding Rnas

Thanks to an international effort through the FANTOM (fantom.gsc.riken.jp) and the ENCODE (encodeproject.org) projects, we know that the non-coding sequences cover 98% of the human genome and that the transcribed part alone covers 90%. Within these RNA sequences that lack protein-coding capacities, lncRNAs are any expressed RNAs of more than 200 nt in length [40,41]. The current classification of lncRNAs gathers five categories, according to their original genomic location in regards to their corresponding protein-coding gene. These five categories are • intergenic, • intronic, • sense, • antisense, and • bidirectional [42]. According to the NONCODE (noncode.org) database file “NONCODEv5_human_hg38_lncRNA.gtf”, 172,216 lncRNA transcripts can theoretically be found in humans [43]. From our literature screening in January 2020, we found hundreds of them potentially associated with COPD, asthma, IPF, or lung cancer. This review focuses on the lncRNAs commonly found between these four lung diseases. While we will summarize their mechanisms of action regarding these diseases, additional studies may also help to understand the full picture of lncRNAs [42,44,45,46,47].

#### 1.3.1. Transcript-Regulating LncRNAs

Under the mRNA transcript degradation process, mature miRNAs bind to the Argonaute RISC Catalytic Components 1 to 4 (AGO1-4). This complex will then target an mRNA 3’UTR leading to the degradation of the mRNA. However, transcript-regulating lncRNAs (treg-lncRNAs) can prevent such mRNA degradation. The lncRNA can act as an RNA decoy for a miRNA, leading to a • miRNA sequestration, • miRNA degradation, or a • translational repression of the mRNA [48]. The miRNA sequestration and the miRNA degradation are part of the competing endogenous RNA (ceRNA) network, which aims at circumventing miRNAs from their original targets [49]. The sequestration controls the miRNA abundance in the cell and inhibits its activity [41]. This process also called “miRNA sponge”, happens just before a miRNA could regulate its target mRNAs through a physical binding between the lncRNA and the miRNA. More precisely, the miRNA binding site, located in the 3’UTR of the lncRNA, does not allow for the degradation of the lncRNA, as it would occur for protein-coding transcripts. Instead, mismatched nucleotides in the lncRNA binding site lead to sequestration of the miRNA by the lncRNA. Subsequently, a sufficient amount of lncRNAs would act as a “sponge,” disabling the mRNA regulation by the sequestrated miRNA [41]. Furthermore, one lncRNA can have multiple miRNA targets, and circular lncRNAs can also regulate the miRNA activity [47]. Interestingly, lncRNAs may be involved in positive feedback loops while targeting miRNAs. For example, a recent study by Qu et al. describes the upregulation of lncRNA ZEB1-AS1 observed in NSCLC cells. ZEB1-AS1 can sequester the miRNA hsa-miR-409-3p, which leads to an increase in the mRNA and protein levels of ZEB1. In return, ZEB1 binds the promoter region of ZEB1-AS1 and activates its expression [50].

*NAT-lncRNAs Specific Regulation:* Few mechanisms may be specific to Natural Antisense transcripts (NATs). NATs are RNA sequences that are complementary to and overlap with either protein-coding or non-coding transcripts [42,51]. Cis-NATs are transcribed from the same genomic locus and have a perfect complementarity with their target mRNA transcript. Trans-NATs are transcribed from a different genomic locus and have an imperfect complementarity with their target mRNA transcript [42]. The NATs may act on the transcription through transcriptional interference, RNA masking, and RNA “A to I” editing [42]. NATs may also regulate the abundance of mRNAs by • suppressing the translation through polysome displacing, • promoting the mRNA decay through 3’UTR binding, or • modulating the mRNA stability and increasing its expression level through the formation of sense or antisense pairs [44]. While the majority of the studies described their actions in cis, no study invalidates a possible operation in trans.

#### 1.3.2. Epigenetics-Regulating LncRNAs

Epigenetics-regulating lncRNAs (epi-lncRNAs) are lncRNAs that may guide the polycomb chromatin domains until polynucleosome compaction [42,52]. The Polycomb group of proteins was identified as a transcriptional-repressive complex, named the Polycomb Repressive Complex (PRC). Essential members of the PRC are the PRC1 and PRC2. The PRC1 contains the CBX7 protein, and the PRC2 contains the EZH1, EZH2, EED, and the SUZ12 subunits. Importantly, the PRC2 is highly conserved between plants and animals and can create polycomb chromatin domains with the PRC1 to help polynucleosome compaction [53]. Indeed, PRC1’s CBX7 and PRC2’s EZH2-SUZ12 can tether epi-lncRNAs, which in return will guide the polycomb chromatin domain through its repressive action. Then, PRC2’s EZH2 and EZH1 trimethylates the histone 3 at lysine 27 (H3K27me3), which will become an anchor site for the PRC1. Once the PRC1 is attached, it mono-ubiquitinates the H2A on K119 (H2AK119Ub), eventually leading to the repression of the targeted gene [42,53]. However, the PRC2 may tether many types of RNA without a precise binding site. In consequence, assigning only the lncRNAs to the recruitment of the PRC2 may be premature [54]. Moreover, epi-lncRNAs may regulate gene expressions independently [47]. For example, HOTAIR can repress a limited number of genes through H3K27me3 without the involvement of the PRC2 complex [55]. Hence, these recent observations point up the need for a better understanding of the epigenetics modulation triggered by epi-lncRNAs.

Besides, the lncRNAs are generally expressed at low levels when compared to protein-coding genes [56,57]. This low expression underlines a fine regulation of their target mRNA, which could dramatically impact the behavior of the receiving cell during intercellular communication. However, the mechanisms are not clear enough to understand how an epi-lncRNAs, which acts in the nucleus, can reach its targets in another cell through vesicle transportation.

## 2. LncRNAs and Their Exosomes in Lung Diseases

From a PubMed screening, done in January 2020, we found associations only between lung cancers and both lncRNAs and exosomes. Therefore, our strategy was first to build a list of the most published lncRNAs found in at least two lung diseases within asthma, COPD, IPF, and lung cancers. Importantly, this information comes from either the main text, the figures, or the gene expression datasets that were available. After overlapping the official gene symbols, we found the ten following lncRNAs in at least two diseases: H19, MALAT1, MEG3, FENDRR, CDKN2B-AS1, TUG1, HOTAIR, GAS5, LINC00861, and CCDC18-AS1. Interestingly, we did not find publications reporting LINC00861 and CCDC18-AS1 in lung cancers.

**Figure 2:**
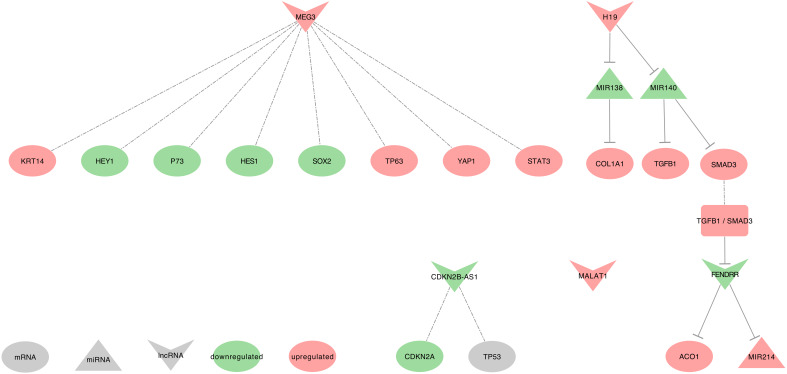
The selected eight lncRNAs in Idiopathic Pulmonary Fibrosis (IPF): A network of lncRNA - miRNA - Protein interactions. Upregulated molecules are in green. Downregulated molecules are in red. Up or down-regulated molecules are in purple. Shape definitions are in grey. Gray arrows target the activated molecules. T-ended lines target the inhibited molecules. Square-ended lines represent a binding between molecules. Dashed-dot lines represent an association with unknown interaction.

**Figure 3:**
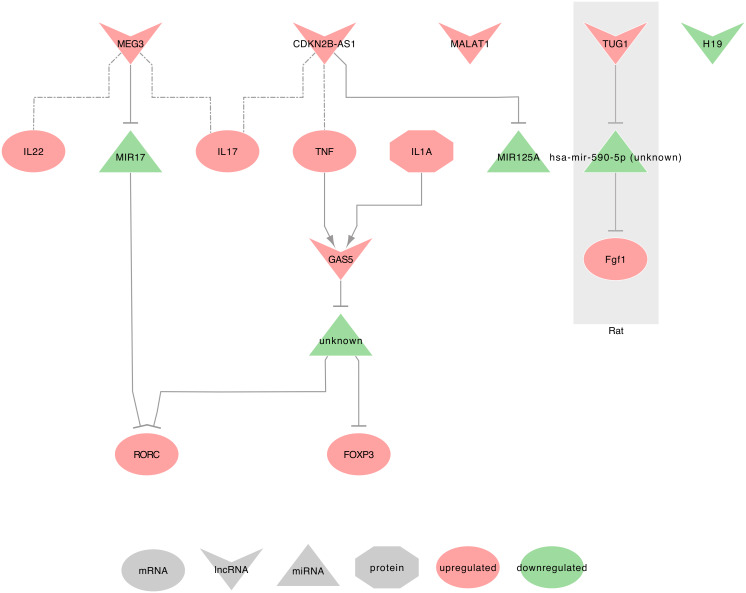
The selected eight lncRNAs in Asthma: A network of lncRNA - miRNA - Protein interactions. Upregulated molecules are in green. Downregulated molecules are in red. Up or down-regulated molecules are in purple. Shape definitions are in grey. Gray arrows target the activated molecules. T-ended lines target the inhibited molecules. Square-ended lines represent a binding between molecules. Dashed-dot lines represent an association with unknown interaction.

**Figure 4:**
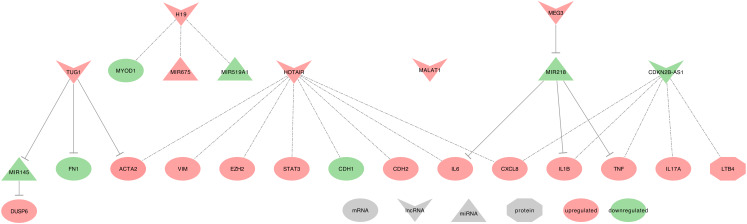
The selected eight lncRNAs in Chronic Obstructive Pulmonary Disease (COPD): A network of lncRNA - miRNA - Protein interactions. Upregulated molecules are in green. Downregulated molecules are in red. Up or down-regulated molecules are in purple. Shape definitions are in grey. Gray arrows target the activated molecules. T-ended lines target the inhibited molecules. Square-ended lines represent a binding between molecules. Dashed-dot lines represent an association with unknown interaction.

Hereafter we will describe the ten lncRNAs in their associated disease. We will then summarize the eight lncRNAs found in lung cancers into interaction networks and tables listing their promising clinical interests. Figure 2, Figure 3 and Figure 4 summarize these lncRNAs and their known actions in the COPD, IPF, and asthma, respectively. Dedicated networks will then cover each of these lncRNAs in lung cancers. Table 1 and Table 2 respectively provide the downstream targets of the eight lncRNAs and their possible use as biomarkers in the lung diseases mentioned above. Table 3 provides lung cancer treatment resistances associated with the eight lncRNAs. Moreover, from this list, only H19, MALAT1, HOTAIR, and GAS5 were associated with exosomes and lung cancers in our PubMed search. Therefore, we will also shortly describe results on exosomes for six additional lncRNAs that were related to only lung cancers. These lncRNAs are UCA1, lnc-MMP2-2, GAPLINC, TBILA, AGAP2-AS1, and SOX2-OT.

### 2.1. H19

H19 Imprinted Maternally Expressed Transcript (H19) is an RNA gene localized on the cytogenetic band 11p15.5, in an imprinted region and is close to the IGF2 gene. H19 is maternally-imprinted, whereas IGF2 is paternally-imprinted. H19 gene has 12 transcripts, 3 retained introns, and 9 lncRNAs [122,123,124]. Interestingly, in human adrenocortical carcinoma cell lines (NCI-H295R), the induction of H19 gene expression comes along with a decrease of IGF2 expression, suggesting a direct mRNA expression regulation [125].

#### 2.1.1. H19 and IPF

In human pulmonary fibrotic tissues from IPF patients, H19 is upregulated and induces fibrosis using the TGFB/SMAD3 signaling pathway, through hsa-miR-140 sequestration. Indeed, the hsa-miR-140 can repress the mRNA and protein expressions of TGFB1 and phospho-SMAD3 [65]. Moreover, in TGFB1 induced fibroblasts, H19 upregulation releases COL1A1 expression through hsa-miR-196a (hsa-miR-196a-1 or hsa-miR-196a-2) sequestration, thus leading to increase cell proliferation and migration [126].

#### 2.1.2. H19 and Asthma

Austin et al. found H19 downregulated in airway smooth muscle (ASM) cells from non-severe asthma when compared to healthy patients. Please refer to their microarray experiment in supplemental Table 10 [127]. However, the authors did not focus on H19 in their study, which requires further efforts to assess its clinical impact and mechanism of action.

#### 2.1.3. H19 and COPD

H19 is upregulated in the quadriceps of the low fat-free mass index (FFMI) COPD patients when compared to normal FFMI. H19 hosts the hsa-miR-675, a miRNA also upregulated in low FFMI COPD patients. The increase of H19 expression may be the consequence of an altered methylation of its region. Besides, H19 expression is associated with the downregulation of MYOD1 and hsa-miR-519a (hsa-miR-519a-1 or hsa-miR-519a-2) in male patients with severe COPD [68]. Subsequently, we can suspect that H19 sequester hsa-miR-519a following demethylation, thus contributing to increase the susceptibility to a low FFMI for the COPD patients.

#### 2.1.4. H19 and Lung Cancer

**Figure 5:**
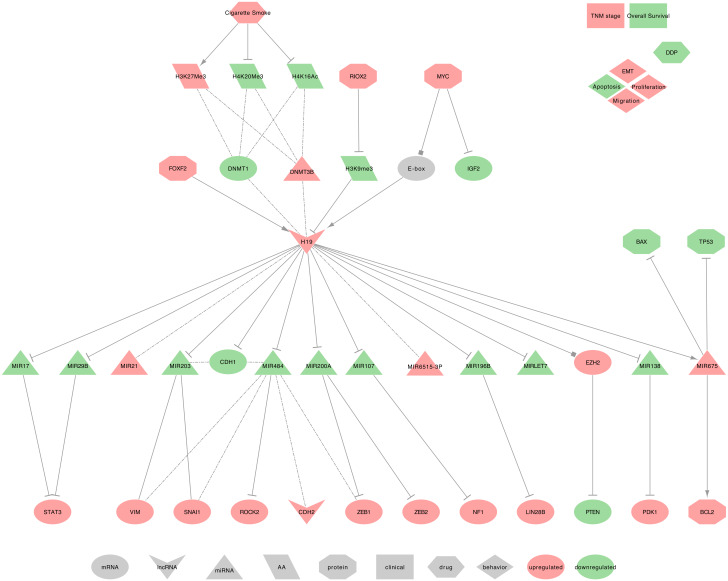
H19 in lung cancers: A network of lncRNA - miRNA - Protein interactions. Upregulated molecules are in green. Downregulated molecules are in red. Shape definitions are in grey. Gray arrows target the activated molecules. T-ended lines target the inhibited molecules. Square-ended lines represent a binding between molecules. Dashed-dot lines represent an association with unknown interaction.

Several studies described H19 as upregulated in NSCLC cells and tumors tissues. They demonstrated that H19 induces cell proliferation, migration, viability, invasion, and epithelial-mesenchymal transition (EMT) while decreasing apoptosis [128,129,130,131,132,133,134]. H19 uses the following mechanisms, as summarized in Figure 5.

⋄ **Regulators of H19:** In NSCLC tumor tissues, FOXF2 can bind the promoter of H19 and can increase its expression, causing a PTEN downregulation [135]. Additionally, H19 may be a direct transcriptional target of and is induced by MYC in NSCLC tumor tissues. Indeed, MYC binds to H19 promoter’s E-boxes to facilitate histone acetylation and transcriptional initiation. Furthermore, MYC can downregulate the expression of IGF2 independently [136,137]. Besides, Shahdoust et al. found H19 among the seven most differentially expressed lncRNAs in the human airway epithelium of cigarette smokers when compared to non-smokers [138]. Similar results, obtained in cdk-4/hTERT-immortalized human bronchial epithelial cells (HBEC), described the H19 upregulation following prolonged CSC exposure. Interestingly, the same authors also found a general diminution of H4K16ac and H4K20me3 and an overall increase of H3K27me3 levels [74]. Nevertheless, H19 methylation status remains low, as RIOX2 can remove methyl groups from H3K9me3 on the H19 promoter, leading to de-repress H19 transcription [139]. Importantly, Liu et al. suggested that H19 demethylation may precede the methylations that silence tumor suppressor genes such as p16-CDKN2A, MGMT, DAPK, E-cadherin (CDH1), and CDH13 [74]. Moreover, the histone alterations coincided with a decreased DNMT1 and an increased DNMT3B expressions, as well as the activation of the WNT/*β*-catenin signaling pathway during prolonged CSC exposure [74]. Indeed, the authors found that WNT ligands, such as WNT2, WNT5A, WNT6, and WNT10A, and the Wnt signaling targets FOXN1 and TCF7, were up-regulated [74].
⋄ **H19 regulated genes:** In NSCLC tumor tissues, H19 decreases the expression of CDH1 by inducing its promoter methylation and also increases CDH2 and VIM expressions [134,140]. Moreover, in human NSCLC cell lines (A549), H19 may regulate metastasis through the modulation of cell proliferation and cell adhesion proteins, including MACC1, EGFR, β-catenin (CTNNB1), ERK1 (MAPK3) and ERK2 (MAPK1) [141].
⋄ **H19 recruits the PRC2:** In NSCLC tumor tissues, H19 can recruit EZH2 to repress PTEN expression, thus increasing cell proliferation [135].
⋄ **H19 as ceRNA:** Among its known functions, H19 was reported as a heavy miRNA regulator. Indeed, H19 can sequester: • hsa-miR-107, to release the expression of NF1 in NSCLC tumor tissues [128] • hsa-miR-200a, to release the expression of ZEB1 and ZEB2 in NSCLC tumor tissues [130] • hsa-miR-29b-3p and hsa-miR-17, to release the expression of STAT3 in NSCLC tumor tissues [131,132] • hsa-miR-196b to release the expression of LIN28B and induce cell growth in NSCLC tumor tissues [142] • hsa-miR-138 (MIR138-1, MI0000476 or MIR138-2, MI0000455) to release the expression of PDK1 in NSCLC tumor tissues [129] • hsa-miR-484 to release the expression of ROCK2 and increase the levels of phosphorylated JUN as well as the mesenchymal markers N-cadherin (CDH2), vimentin (VIM), ZEB1 and SNAI1 while decreasing the level of the epithelial marker CDH1 in NSCLC tumor tissues and cell lines [133,134,143]. A similar observation can be made with the downregulation of hsa-miRNA-203 (hsa-miRNA-203a or hsa-miRNA-203b), which was associated with VIM and SNAI1 upregulation and CDH1 downregulation in NSCLC tumor tissues [143].
⋄ **19 as a miRNA regulator:** In NSCLC tumor tissues, H19 induces hsa-miR-675-5p expression, which, in turn, increases the expression of BCL2 and decreases the expression of TP53 as well as BAX [144,145,146]. Xu et al. hypothesized H19 as an epigenetic regulator of hsa-miR-6515-3p, which contributes to metastasis [147]. H19 expression was also positively correlated to hsa-miR-21 expression [97].
⋄ **H19 at the clinical level:** H19 upregulation in NSCLC tumor tissues was associated with advanced tumor–node–metastasis (TNM) stages and negatively correlated with patient Overall Survival (OS) [96,97]. H19 expression was also higher in stage III and IV NSCLC, while hsa-miR-21 expression was higher in stage I and II NSCLC when compared to non-tumor lung tissues [97]. Besides, plasma levels of H19 were significantly increased in NSCLC patients when compared to patients with benign lung disease [89]. Additionally, a nucleotide polymorphism, the H19-rs217727 C>T, was found significantly associated with an increased risk of lung cancer [84]. Consequently, with further efforts to confirm these results in large independent cohorts, H19 would make a great biomarker to diagnose or to assess a genetic predisposition to lung cancers.
⋄ **H19 behavior against treatment:** Wang et al. found a negative correlation between the H19 upregulation in NSCLC tumor tissues and the Cisplatin (DDP) response [96].
⋄ **Exosomal H19:** Tumor-released lncRNA H19 (exo-H19) can promote gefitinib resistance via packaging into exosomes in NSCLCs. While the experiments were on gefitinib-resistant NSCLC cell lines, the authors assessed the expression of H19 in both gefitinib-resistant and parental sensitive cells. H19 expression was increased in gefitinib-resistant NSCLC cells and was described as secreted through the incorporation into exosomes, which was mediated by HNRNPA2B1. Moreover, exosome-mediated transfer of H19 conferred gefitinib resistance to the recipient NSCLC cells [148].

### 2.2. MEG3

Maternally Expressed 3 (MEG3) is a maternally expressed imprinted RNA gene localized on the cytogenetic band 14q32.2, in the DLK1-DIO3 imprinted region. MEG3 gene has 50 transcripts, all identified as lncRNAs. MEG3 is expressed in many tissues under normal conditions and interacts with TP53 [149].

#### 2.2.1. MEG3 and IPF

In pulmonary epithelial cells from IPF lung tissue, MEG3 upregulation promotes migration with an upregulation of TP63, keratin 14 (KRT14), STAT3, and YAP1 as well as a downregulation of TP73, SOX2, HES1, and HEY1 [66].

#### 2.2.2. MEG3 and Asthma

In CD4${}^{+}$T-cells of asthmatic patients, MEG3 is upregulated when compared to healthy patients. Moreover, it displays pro-inflammatory properties linked to the increase of Th17 associated cytokines IL17A and IL22. It acts through hsa-miR-17 sequestration, which in turn releases the expression of RORγt (RORC), leading to an increase of Treg/TH17 [59].

#### 2.2.3. MEG3 and COPD

In lung tissues from COPD patients and cigarette smoke extract (CSE)-treated 16HBE cells, MEG3 is upregulated [70,72]. It induces apoptosis and inflammation by releasing inflammatory cytokines IL1β (IL1B), IL6 and TNF α (TNF) expression through hsa-miR-218 (hsa-miR-218-1 or hsa-miR-218-2) sequestration [70].

#### 2.2.4. MEG3 and Lung Cancer

**Figure 6:**
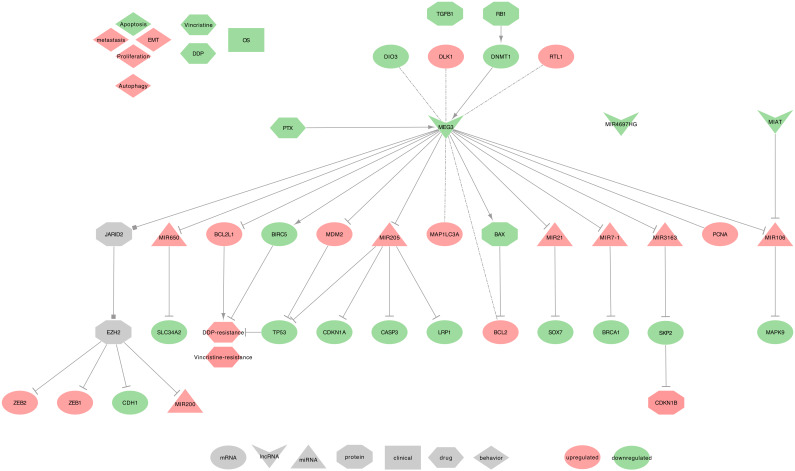
MEG3 in lung cancers: A network of lncRNA - miRNA - Protein interactions. Upregulated molecules are in green. Downregulated molecules are in red. Shape definitions are in grey. Gray arrows target the activated molecules. T-ended lines target the inhibited molecules. Square-ended lines represent a binding between molecules. Dashed-dot lines represent an association with unknown interaction.

Downregulated MEG3 can regulate cell proliferation, EMT, and apoptosis in NSCLC tumor tissues from patients with an advanced pathological stage, as well as in cell lines. Importantly, MEG3 was more downregulated in stages III+IV when compared to stages I+II, and it also increases cell viability and proliferation, while reducing the expression of autophagy [120]. Conversely, MEG3 was significantly less methylated in the tumor of smoker patients with clinical early-stage NSCLC, as compared to non-cancerous tissue. This MEG3 demethylated region (DMR) was associated with a hypermethylated DIO3 and two hypomethylated (DLK1 and RTL1) [150]. Subsequently, the low MEG3 expression observed in NSCLC patients with an advanced pathological stage may be due to a deletion of the MEG3-DMR locus or could be due to the deletion of a transcription factor binding MEG3 promoter [150,151,152]. MEG3 uses the following mechanisms, as summarized in Figure 6.

⋄ **Regulators of MEG3:** MEG3 promoter methylation was reported in 96% of NSCLC tumor tissues, which mainly contributes to its downregulation [153,154]. Alternatively, phosphorylated RB1 has been described to activate DNMT1, which in turn, will also decrease the methylation of the MEG3 locus [155].
⋄ **MEG3 regulated genes:** Two studies realized in NSCLC tumor tissues described MEG3 as an activator of TP53 by decreasing the levels of MDM2 [149,153]. Additionally, MEG3 can decrease BCL2 expression by promoting BAX, and also decrease MAP1LC3A expression [156,157]. Besides, Su et al. described MEG3 as inversely correlated with PCNA [153].
⋄ **MEG3 recruits the PRC2:** In NSCLC cell lines, MEG3 contributes to the recruitment of PRC2’s EZH2, through a possible interaction with JARID2. This PRC2 recruitment will eventually repress the expression of CDH1, ZEB family (ZEB1 and ZEB2), and hsa-miR-200 family ( MIR200A, MIR200B, and MIR200C ), which will lead to a decreased EMT [158].
⋄ **MEG3 as ceRNA:** Among its known functions, MEG3 was reported as a heavy miRNA regulator. Indeed, MEG3 can sequester: • hsa-miR-650 to release the expression of SLC34A2, in NSCLC cell line (H1299) [159] • hsa-miR-7-5p to release the expression of BRCA1, in NSCLC tumor tissues and BEAS-2B, A549, and HCC823 cell lines [156] • hsa-miR-21-5p to release the expression of SOX7, in DDP-resistant NSCLC tumor tissues and A549 and H1299 cell lines [117] • hsa-miR-3163 to release the expression of SKP2 that will, in turn, promotes the ubiquitination-associated degradation of p27 (CDKN1B), in NSCLC tumor tissues and A549 cell lines [153] • hsa-miR-205-5p to release the expression of LRP1. hsa-miR-205-5p may also be involved in the inhibition of TP53, p21 (CDKN1A), and caspase-3 (CASP3) expressions, in NSCLC tumor tissues and MEG3-knockdown NSCLC cell lines [154,157,160]. Moreover, using lncRNA-miRNA-mRNA regulatory network modules, Li et al. showed the following interactions in LUAD tumors from The Cancer Genome Atlas (TCGA). MEG3 and MIAT may interact with hsa-miR-106 (hsa-miR-106a or hsa-miR-106b), which then would regulate the expression of MAPK9. For a full overview of the Lung Adenocarcinoma (LUAD) miRNAs-lncRNAs-mRNAs network, please report to Li et al. Figure 2 [161].
⋄ **MEG3 at the clinical level:** MEG3 low-expression in NSCLC tumor tissues was associated with short-term survival in two independent public datasets [106]. Besides, MEG3 genotype rs4081134 SNP (AA) was associated with a lung cancer risk in Chinese patients [85]. While being promising, these findings should, however, be confirmed in additional large independent cohorts to classify MEG3 as a reliable biomarker.
⋄ **MEG3 behavior against treatment:** MEG3 can promote NSCLC cell lines sensitivity (A549 and H292) to Vincristine, by inhibiting autophagy. Indeed, autophagy level was higher in resistant cells, and the overexpression of MEG3 significantly reduced the expression of autophagy-related proteins LC3-I (MAP1LC3A), and LC3-II (MAP1LC3B) were [120]. The overexpression of MEG3 can also increase the DDP-sensitivity of NSCLC cell lines (A549 and H1299) [117] and xenografts [118] by decreasing TP53, CTNNB1, survivin (BIRC5), therefore targeting the WNT/β-catenin signaling pathway [118], and Bcl-xl (BCL2L1) [119]. Furthermore, in Xu et al., the authors showed that Paclitaxel (PTX) could upregulate MEG3 and TP53, thus inhibiting cell proliferation and promoting the death of A549 cells [162].
⋄ **Exosomal MEG3:** From the PubMed screening, we found no studies on MEG3 associated with lung diseases and exosomes. However, three recent studies could describe exosomal MEG3 in high-grade serous carcinoma [163], cervical cancer [164], and Hunner-type interstitial cystitis [165]. These studies underline that MEG3 may be involved in intercellular communication, especially in cancers, and therefore further research on this topic is needed to assess its relevance in lung diseases.

### 2.3. MALAT1

Metastasis Associated Lung Adenocarcinoma Transcript 1 (MALAT1) is an RNA gene localized on the cytogenetic band 11q13.1. MALAT1 gene has 17 transcripts, all identified as lncRNAs. MALAT1 may also act as a transcriptional regulator for numerous genes and is involved in the cell cycle regulation and pre-mRNA splicing [166].

#### 2.3.1. MALAT1 and Asthma

In Zhu et al. RNA sequencing data, MALAT1 was observed as upregulated in the blood of highly-expressed IgE eosinophilic asthmatic (EA) patients when compared to healthy patients [61]. The authors denoted the T cell receptor signaling as one of the main pathways impaired in EA patients. Moreover, Qiu et al. heatmap (Figure 2A) displays MALAT1 as upregulated in CD4${}^{+}$ cells from asthmatic patients when compared to healthy patients [59].

#### 2.3.2. MALAT1 and COPD

MALAT1 was found the most abundant lncRNA in whole blood cells from former and current smokers with COPD [167].

#### 2.3.3. MALAT1 and Lung Cancer

**Figure 7:**
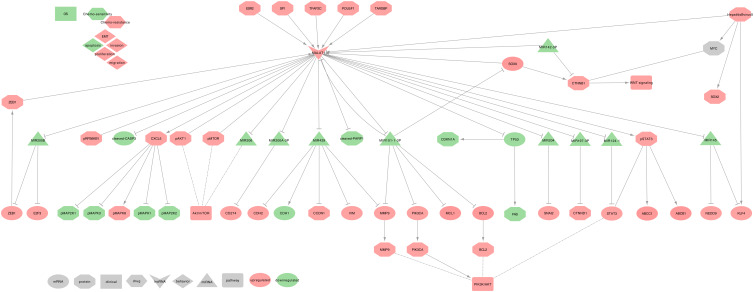
MALAT1 in lung cancers: A network of lncRNA - miRNA - Protein interactions. Upregulated molecules are in green. Downregulated molecules are in red. Shape definitions are in grey. Gray arrows target the activated molecules. T-ended lines target the inhibited molecules. Square-ended lines represent a binding between molecules. Dashed-dot lines represent an association with unknown interaction.

MALAT1 has been described as upregulated in NSCLC tumor tissues and cell lines, and it regulates cell proliferation, migration, invasion, EMT and apoptosis, but also mesenchymal-epithelial transition (MET) [77,79,101,102,168,169,170,171,172,173,174,175,176,177,178]. It is worth underlining that Ghafouri-Fard et al. summarized the studies of MALAT1 to a broader range of cancers in the Table 1 of their review [179]. MALAT1 uses the following mechanisms, as summarized in Figure 7.

⋄ **Regulators of MALAT1:** TDP-43 (TARDBP) can upregulate MALAT1 expression through direct interaction in NSCLC cell lines (A549 and YTLMC-9) [169]. Moreover, in the tumor tissues of NSCLC female patients, ESR2 can upregulate MALAT1 expression by binding to estrogen-response-element I and II on the proximal 2-kb region of MALAT1 promoter [172]. Additionally, in the NSCLC cell line (A549), Oct3/4 (POU5F1) and SP1 can increase MALAT1 expression by physically binding its promoter [178,180]. Furthermore, in NSCLC cell lines (SPC-A1 and H1299) in vitro and in vivo, TFAP2C and ZEB1 can upregulate MALAT1 expression, leading to the sequestration of miR-200b, which, in turn, increases of E2F3 and ZEB1, creating, therefore, a positive feedback loop [181]. Some miRNAs may also regulate MALAT1. Indeed, hsa-miR-142-3p can inhibit MALAT1 and WNT/β-catenin signaling pathway in NSCLC tumor tissues and H1299 cell lines [75]. Moreover, hsa-miR-101-3p can inhibit MALAT1, BCL2, MMP9, PI3K (PIK3CA) expressions in NSCLC cell lines (H1299 and H520) [77].
⋄ **MALAT1 regulated genes:** In NSCLC cell lines (A549 and H1299), MALAT1 can repress TP53 expression at the pre-mRNA level by binding a responsive region in the TP53 P1 promoter, leading to the downregulation of CDKN1A and FAS expressions [170]. In NSCLC cell lines (PC-9 and A549), MALAT1 also decreases cleaved-PARP1, cleaved-CASP3, and upregulates phospho-STAT3 [171], which in turn upregulates MDR1 (ABCB1) and MRP1 (ABCC1) [182]. Moreover, MALAT1 is involved in the upregulation of BCL2, MMP9, PIK3CA expressions, thus activating the PI3K/AKT signaling pathway in NSCLC tumor tissues and H1299 cell lines [77]. Furthermore, in NSCLC tumor tissues and cell lines (A549 and H1299), MALAT1 can upregulate VIM and downregulate CDH1 and is involved in the phosphorylation of AKT1, RPS6KB1, and MTOR [79,102]. Besides, in NSCLC tumor tissues and cell lines (A549, H661, and H460), MALAT1 can upregulate CXCL5, which in turn upregulates p-MAPK8 and down-regulates p-MAP2K1/2, p-MAPK3/1 proteins [176,183].
⋄ **MALAT1 as ceRNA:** Among its known functions, MALAT1 was reported as a heavy miRNA regulator. Indeed, MALAT1 can sequester: • hsa-miR-145 to release the expressions of NEDD9 in the tumor tissues of NSCLC female patients [172], and KLF4 in NSCLC tumor tissues and cell lines (A549 and H1299) [184] • hsa-miR-204 to release the expression of SNAI2 in NSCLC tumor tissues and cell lines (A549, H1299, H460, and H446) [185] • hsa-miR-124-1 to release the expression of STAT3 and PI3K/AKT signaling pathway, in NSCLC cell lines (A549, H23, H522, H1299, H460) [78] • hsa-miR-206 and impact the AKT/MTOR signaling pathway in NSCLC tumor tissues and cell lines (A549 and H1299) [79] • hsa-miR-200b to release E2F3 and ZEB1 expressions, thus creating a positive feedback loop as ZEB1 can upregulate MALAT1 in NSCLC cell lines (SPC-A1 and H1299) in vitro and in vivo [181] • hsa-miR-200a-3p to release PD-L1 (CD274) expression in NSCLC tumor tissues and cell lines (A549 and CAL-12T) [186] • hsa-miR-429 to release Cyclin D1 (CCND1), MMP9, VIM, and CDH2 expression while repressing CDH1 expression in NSCLC tumor tissues [187] • hsa-miR-197-3p to release the Catenin-δ1 (CTNND1) expression in NSCLC tumor tissues and cell lines (A549, H1299, H460 and SPC-A-1) [188] • hsa-miR-101-3p to release the expressions of MCL1 in NSCLC tumor tissues and cell lines ((A549, H1299, H469, SPC-A1) [103], and SOX9 in NSCLC tumor tissues and cell lines (A549, H1299, HCC827, and H358) [76]. Moreover, in NSCLC tumor tissues and cell lines (A549, H1299, HCC827, and H358), SOX9 could activate MALAT1 expression by binding MALAT1 promoter on a specific site (5′-TCATTGTGT-3′), thus creating a positive feedback loop which dramatically increases MALAT1 downstream effects. Besides, SOX9 contributes to the upregulation of CTNNB1, a downstream target of MYC, thus activating the WNT/β-catenin signaling pathway [76].
⋄ **MALAT1 at the clinical level:** Several studies described a high MALAT1 level associated with a poor prognosis and short OS in NSCLC tumor tissues [76,79,101,102,103,105]. Besides, MALAT1 level was low in the serum of patients with NSCLC; however, it still lacks some specificity [90,91,92,93]. Furthermore, both MALAT1 and SOX9 expressions were associated with age, tumor size, and TNM stage, making these two genes potential candidates for prognosis tools [76,79,104].
⋄ **MALAT1 behavior against treatment:** High MALAT1 expression was associated with a DDP chemo-resistance in NSCLC tumor tissues [76,103,184,188] and cell lines [182]. Interestingly, a feedback loop between MALAT1 and SOX9 may amplify this resistance [76]
⋄ **Exosomal MALAT1:** Exosomal MALAT1 (exo-MALAT1) was described as upregulated in the serum of 77 NSCLC patients, and its expression was related to tumor stage and lymphatic metastasis [174]. While being a potential new biomarker for tumor stage diagnosis, further studies should be conducted on larger cohorts to confirm the predictive power of exo-MALAT1.

### 2.4. FENDRR

FOXF1 adjacent non-coding developmental regulatory RNA (FENDRR) is an RNA gene localized on the cytogenetic band 16q24.1. FENDRR gene has 14 transcripts, all identified as lncRNAs. This gene is transcribed bidirectionally with FOXF1 on the opposite strand. FENDRR may bind the polycomb repressive complex 2 (PRC2) to promote the methylation of its targeted genes.

#### 2.4.1. FENDRR and IPF

FENDRR levels were low in fibrotic human lung cells and mouse primary lung fibroblasts. Interestingly, Huang et al. hypothesized that the TGFB1/SMAD3 signaling pathway might cause these low levels. Besides, FENDRR may inhibit fibroblast activation and reduces pulmonary fibrosis by capturing ACO1, thus reducing the iron levels, and by sequestrating the profibrotic hsa-miR-214 [64].

#### 2.4.2. FENDRR and Lung Cancer

**Figure 8:**
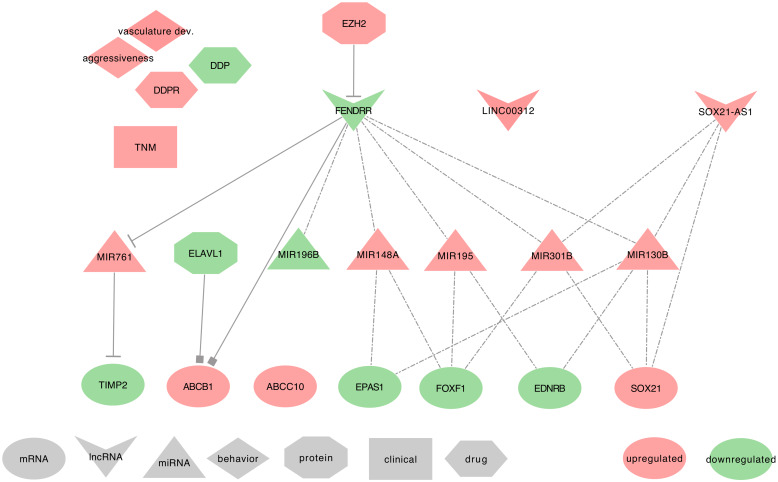
FENDRR in lung cancers: A network of lncRNA - miRNA - Protein interactions. Upregulated molecules are in green. Downregulated molecules are in red. Shape definitions are in grey. Gray arrows target the activated molecules. T-ended lines target the inhibited molecules. Square-ended lines represent a binding between molecules. Dashed-dot lines represent an association with unknown interaction.

Several studies described FENDRR as downregulated in NSCLC tumor tissues and cell lines. They also found FENDRR within the top three lncRNA sharing high connectivity with differentially expressed protein-coding genes. Besides, network prediction algorithms associated FENDRR with vasculature development, cell surface receptor-linked signal transduction, cell proliferation, EMT, stemness, metastasis, and apoptosis [88,116,189,190,191,192,193,194]. FENDRR uses the following mechanisms, as summarized in Figure 8.

⋄ **Regulators of FENDRR:** Two studies described FENDRR as hypermethylated through a presumable involvement of EZH2. This hypermethylation could explain its downregulation in NSCLC tumor tissues and cell lines (Calu-1 and H1975) [193,194].
⋄ **FENDRR regulated genes:** In NSCLC tumor tissues, FENDRR can specifically bind to the 3’UTR of ABCB1, thus blocking HuR (ELAVL1) binding to ABCB1 3’UTR, and therefore resulting in the decrease of ABCB1 expression [195]. Besides, Xu et al. found a negative correlation between FENDRR and ABCC10 expressions in NSCLC tumor tissues and cell lines (A549) [116].
⋄ **FENDRR as ceRNA:** In NSCLC tumor tissues, cell lines (H1650, HCC827, A549, and H1975) and xenografts, FENDRR could also act as a miRNA regulator. Indeed, FENDRR can sequester hsa-miR-761 to release the expression of the metalloproteinase inhibitor TIMP2 [189,196]. Additionally, Liu et al. associated FENDRR to prognostic-significant ceRNA networks using TCGA-LUAD data. They also listed seven other molecules associated with these ceRNA networks; three mRNAs, EPAS1, FOXF1, and EDNRB, and four miRNAs, hsa-miR-148a, hsa-miR-195, hsa-miR-196b, and hsa-miR-301b). For an exhaustive overview of their FENDRR centered lncRNA–miRNA–mRNA ceRNA network, please refer to Figure 5 of their study [197].
⋄ **FENDRR at the clinical level:** FENDRR low-expression in tumor tissues is strongly associated with TNM 1 stage in LUAD patients. Furthermore, when associated with LINC00312, FENDRR showed a diagnostic value in detecting these LUAD patients [88]. Nevertheless, to confirm the diagnostic power of FENDRR on the TNM 1 stage of LUAD patients, additional studies should be conducted on a broader spectrum of lung cancers, including different cancer subtypes and TNM stages.
⋄ **FENDRR behavior against treatment:** The low expression of FENDRR observed in NSCLC tumor tissues was correlated with chemo-resistance to DDP [116]. However, additional studies are required to confirm this result.
⋄ **Exosomal FENDRR:** From the PubMed search, we found no studies associating FENDRR to exosomes. However, FENDRR is an important lncRNA that controls the occurrence of metastasis. Indeed, low FENDRR expression was associated with distant metastasis and allowed the downregulation of the metalloproteinase inhibitor TIMP2 by the lack of competition with hsa-miR-761. Therefore, the metalloproteinase can degrade the extracellular matrix (ECM) and facilitates tumor metastasis [196]. It would, therefore, be interesting to seek for FENDRR in the extracellular vesicles of lung cancer associated with distant metastasis.

### 2.5. TUG1

Taurine Up-Regulated 1 (TUG1) is an RNA gene localized on cytogenetic band 22q12.2. TUG1 gene has 20 transcripts, all identified as lncRNAs. TUG1 interacts with the PRCs to regulate the transcription and may also act as a ceRNA targeting miRNAs.

#### 2.5.1. TUG1 and Asthma

In ASM of Sprague Dawley rats, rat TUG1 was described upregulated and release rat Fgf1 through rno-miR-590-5p sequestration, thus leading the increase of cell proliferation and migration [62].

#### 2.5.2. TUG1 and COPD

Gu et al. found TUG1 as upregulated, in sputum and lung tissues from COPD patients with or without a smoking history. They also demonstrated that TUG1 releases the expression of DUSP6 through the sequestration of hsa-miR-145-5p, thus contributing to the inhibition of inflammation and airway remodeling [71]. Besides, in TGFB1 treated BEAS-2B and HFL1 cells, TUG1 could block cell proliferation through the inhibition of αSMA (ACTA2) and fibronectin 1 (FN1) expressions [72].

#### 2.5.3. TUG1 and Lung Cancer

**Figure 9:**
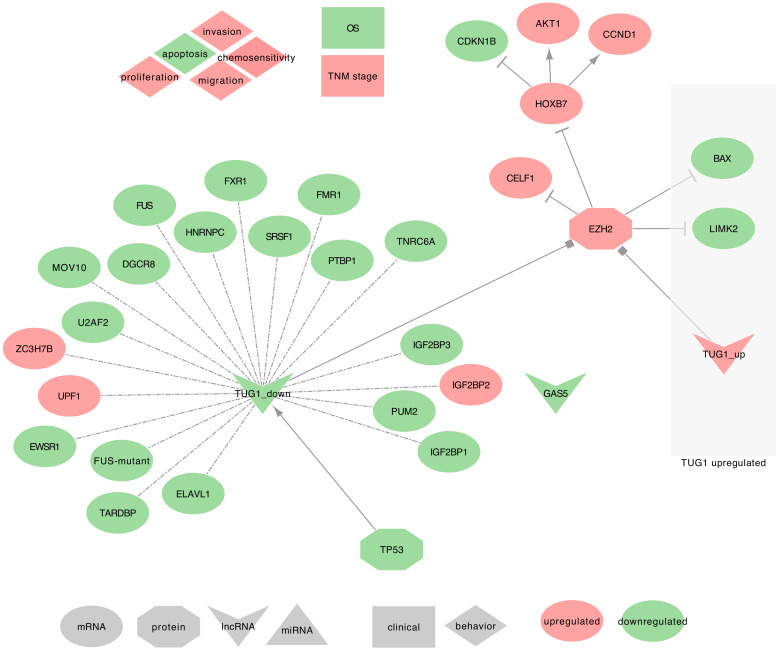
TUG1 in lung cancers: A network of lncRNA - miRNA - Protein interactions. Upregulated molecules are in green. Downregulated molecules are in red. Shape definitions are in grey. Gray arrows target the activated molecules. T-ended lines target the inhibited molecules. Square-ended lines represent a binding between molecules. Dashed-dot lines represent an association with unknown interaction.

TUG1 was the only lncRNA described as downregulated in NSCLC tumor tissues and upregulated in Small Cell lung cancer (SCLC) tissues and the serum of LUAD patients. Interestingly, TUG1 downregulation was significant in the tumor tissues of male donors only and was associated with Squamous Cell Carcinoma (SCC) and LUAD tumor subtypes. This decrease was, however, strongly and significantly correlated to GAS5 decrease in Female donors and combined tumors when compared to adjacent non-cancerous tissues (ANCTs) [95]. Furthermore, few studies described TUG1 associated with cell proliferation, migration, invasion, apoptosis, and autophagy [198,199,200]. It is worth underlining that Ghaforui-Fard et al. summarized the studies of TUG1 to a broader range of cancers in Table 1 of their review [198]. TUG1 uses the following mechanisms, as summarized in Figure 9.

⋄ **Regulators of TUG1:** Zhang et al. found that TP53 can regulate TUG1 expression in NSCLC tumor tissues and SPC-A1 cell line in vitro and in vivo [105,107]. This result requires, however, an independent validation. **⋄ TUG1 regulated genes:** In NSCLC tumor tissues, TUG1 has been described to trans-downregulate the expression of Homeobox B7 (HOXB7), CELF1, and EZH2 (PRC2 subunit). Moreover, TUG1 decrease was also significantly associated with the differential expression of the following target genes. In both LUAD and SCC, TUG1 downregulation was associated with the downregulation of ELAVL1, PTBP1, IGF2BP1, IGF2BP2, IGF2BP3, PUM2, TNRC6A, DGCR8, FMR1, FXR1, FUS, MOV10, ZC3H7B, EWSR1, FUS-mutant, SRSF1, U2AF2, UPF1, and TARDBP [95]. It was otherwise associated with the upregulation of HNRNPC [95]. Conversely, in SCLCs, TUG1 may silence LIMK2 and BAX expression by interacting with EZH2 [86,105,107,108,198,199,200].
⋄ **TUG1 recruits the PRC2:** In NSCLC tumor tissues, TUG1 can regulate CELF1 through PRC2 binding [108], and may also recruit EZH2, which will then trimethylates H3K27 and repress HOXB7 [95].
⋄ **TUG1 as ceRNA:** In NSCLC tumor tissues, cell lines (SPC-A1, NCI-H520, NCI-H520, NCI-H1299) and xenograft, Guo et al. found that TUG1 can sequester hsa-miR-221, thus releasing the expression of PTEN [83].
⋄ **TUG1 at the clinical level:** On the one hand, TUG1 was upregulated in the serum of LUAD patients when compared to healthy serums [86,198]. On the other hand, TUG1 low expression in NSCLC tumor tissues was associated with a high TNM stage and a poor patient outcome [105,107,108,198].
⋄ **TUG1 behavior against treatment:** The low expression of TUG1 observed in NSCLC cells was associated with chemo-resistance to DDP. When overexpressed, TUG1 promoted the sensitivity of NSCLC cells to DDP, leading to apoptosis, in vitro, and in vivo [83].
⋄ **Exosomal TUG1:** From the PubMed search, we found no studies associating TUG1 with lung diseases and exosomes. However, two recent studies mentioned exosomal TUG1, in MCF-7 cells, the levels of TUG1 were moderately elevated in exosomes when compared to cells [201], and TUG1 was up-regulated in the serum exosomes of colorectal cancer (CRC) patients [202]. Besides, Guo et al. described TUG1 as ceRNA to hsa-miR-221, which enables PTEN expression [83]. Together, these results suggest that TUG1 may be involved in intercellular communication to synchronize cellular proliferation. Therefore, further research on this topic is needed to assess its relevance in lung cancers.

### 2.6. CDKN2B-AS1, ANRIL

CDKN2B Antisense RNA 1 (CDKN2B-AS1), also called ANRIL, is an RNA gene localized on cytogenetic band 9p21.3. CDKN2B-AS1 gene has 28 transcripts, all identified as lncRNAs and with some of which can turn into circular RNAs [203]. CDKN2B-AS1 is known to bind CBX7 (PRC1 subunit) and SUZ12 (PRC2 subunit) to repress the transcription of p15 (CDKN2B) [42,204,205].

#### 2.6.1. CDKN2B-AS1 and IPF

In the peripheral blood of IPF patients, CDKN2B-AS1 is downregulated when compared to healthy controls. The adjacent gene, CDKN2A, is transcribed simultaneously with CDKN2B-AS1 and is also downregulated in IPF patients. Importantly, Du et al. described that both CDKN2B-AS1 and CDKN2A might regulate the TP53 signaling pathway [63]. Indeed, the CDKN2A protein is known to stabilize TP53 in NSCLC [206], and the authors found the p53-signaling pathway as the top target gene-associated pathway in IPF patients.

#### 2.6.2. CDKN2B-AS1 and Asthma

In their study on bronchial asthma at exacerbation (BA-E) and bronchial asthma at remission (BA-R), Ye et al. described CDKN2B-AS1 as upregulated in the plasma of patients with both types of bronchial asthma when compared to healthy subjects. Moreover, CDKN2B-AS1 is upregulated in BA-E patients when compared to BA-R patients. These authors also found a negative correlation between CDKN2B-AS1 and hsa-miR-125a in all patients, suggesting miRNA sequestration. In Table 2 and Table 3 from the same study, the authors found CDKN2B-AS1 negatively correlated with FEV_1/FVC in BA-E patients, and positively correlated with pro-inflammatory cytokines, such as TNF in BA-E patients and IL17A in both BA-E and BA-R patients [58].

#### 2.6.3. CDKN2B-AS1 and COPD

Ge et al. described CDKN2B-AS1 as downregulated in the plasma from acute exacerbations of COPD (AECOPD) when compared to stable COPD or healthy patients. These authors also found a negative correlation between the expression of CDKN2B-AS1 and inflammatory cytokines such as TNF, IL1B, IL17A, and Leukotriene B4 (LTB4) in both AECOPD and stable COPD patients. Furthermore, they found another negative correlation between CDKN2B and IL8 (CXCL8) only in AECOPD patients [67].

#### 2.6.4. CDKN2B-AS1 and Lung Cancer

**Figure 10:**
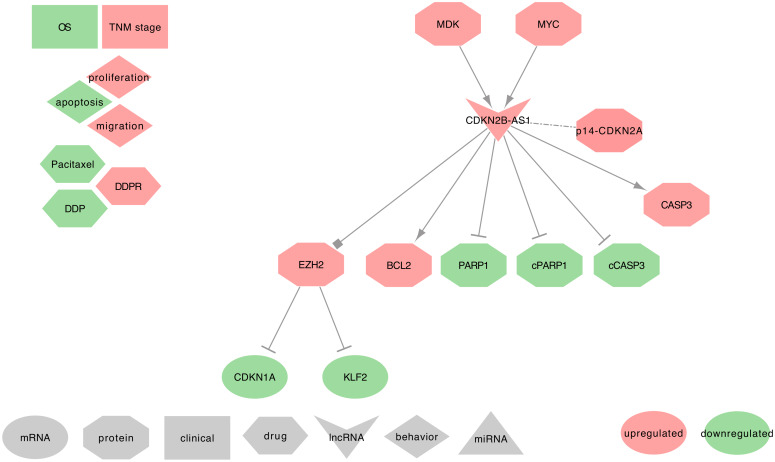
CDKN2B-AS1 in lung cancers: A network of lncRNA - miRNA - Protein interactions. Upregulated molecules are in green. Downregulated molecules are in red. Shape definitions are in grey. Gray arrows target the activated molecules. T-ended lines target the inhibited molecules. Square-ended lines represent a binding between molecules. Dashed-dot lines represent an association with unknown interaction.

CDKN2B-AS1 was described as upregulated in NSCLC tumor tissues and cell lines and was among the top three lncRNAs with high connectivity with differentially expressed protein-coding genes [87,88,207]. CDKN2B-AS1 high expression is also known to promote cell proliferation, cell migration, and to be involved in apoptosis [208,209]. CDKN2B-AS1 uses the following mechanisms, as summarized in Figure 10.

⋄ **Regulators of CDKN2B-AS1:** In NSCLC tumor tissues and cell lines (A549, SPC-A1, and NCI-H1975), MYC can upregulate CDKN2B-AS1 by physically interacting with the c-Myc-responsive element (E-box) of CDKN2B-AS1 promoter [87,208]. Besides, Midkine (MDK) was described as often upregulated in the tumor microenvironment of SCC tumor tissues and cell lines (SCC4, OSCC3, HSC3, and CAL27). The authors also found that MDK can upregulate the CDKN2B-AS1 expression [121].
⋄ **DKN2B-AS1 regulated genes:** In NSCLC tumor tissues, Alsibai et al. found a strong positive correlation between the expressions of CDKN2B-AS1 and the tumor suppressors p15-CDKN2B and p14-CDKN2A, but not p16-CDKN2A. Interestingly, expressed CDKN2B-AS1 can stabilize the PRC complexes to repress the expression of p15, p14, and p16, leading to activate the cell cycle [207]. Few additional studies described the ability of CDKN2B-AS1 to decrease the expression of PARP1, cleaved-PARP1, and cleaved-CASP3, and to increase BCL2 and CASP3 expressions [87,121].
⋄ **CDKN2B-AS1 recruits the PRC2:** CDKN2B-AS1 can also silence KLF2 and CDKN1A transcription by binding with EZH2 in NSCLC tumors tissues and cell lines (PC9, SPC-A1, NCI-H1975, H1299, H358 and (H520) [209].
⋄ **CDKN2B-AS1 at the clinical level:** Lin et al. found a positive correlation between CDKN2B-AS1 high-expression and the differentiation grade and TNM stages in LUAD [87]. CDKN2B-AS1 high-expression in NSCLC tumor tissues was also associated with poor patient OS [94]. Intriguing results from Du et al. suggested that a low CDKN2B-AS1 expression in the peripheral blood of IPF patients may promote the occurrence of lung cancers by regulating the P53 signaling pathway [63]. However, further investigations should be conducted on IPF patients that developed lung cancer to confirm this hypothesis. Interestingly, CDKN2B-AS1 SNPs were strongly associated with the risk of developing a LUAD [210,211,212,213,214].
⋄ **CDKN2B-AS1 behavior against treatment:** CDKN2B-AS1 high expression increases PTX resistance of A549 cells [87]. Interestingly, Zhang et al. showed that cancer-associated fibroblasts (CAFs) contribute to the high level of MDK in the tumor micro-environment of Oral Squamous Cell Carcinoma (OSCC) tissues, thus promoting a DDP resistance via a high expression of CDKN2B-AS1 [121].
⋄ **Exosomal CDKN2B-AS1:** From the PubMed search, we did not find studies associating CDKN2B-AS1 with lung diseases and exosomes. Moreover, only one recent study mentioned exosomal CDKN2B-AS1 as significantly higher in the urine of BC patients when compared to healthy subjects [215]. However, CDKN2B-AS1 is an important lncRNA that can decrease the expression levels of PARP1, which plays a crucial role in DNA repair [87,121]. Since the alteration of the DNA repair mechanism is part of the hallmark of cancers, it would be interesting to seek for CDKN2B-AS1 in the extracellular vesicles of early-stage lung tumors.

### 2.7. HOTAIR

HOX Transcript Antisense RNA (HOTAIR) is an RNA gene localized on the cytogenetic band 12q13.13. HOTAIR gene has five transcripts, all identified as lncRNAs.

#### 2.7.1. HOTAIR and COPD

In Male BALB/c mice exposed for four days with CS as well as human bronchial epithelial (HBE) cells treated with CSE, STAT3 activation led to the upregulation of HOTAIR and EZH2. Additionally, the levels of inflammatory factors, IL6 and CXCL8, as well as the EMT markers, CDH2, VIM, and ACTA2, increased, while CDH1 levels decreased. Nevertheless, these results shall be confirmed in COPD patients [69].

#### 2.7.2. HOTAIR and Lung Cancer

**Figure 11:**
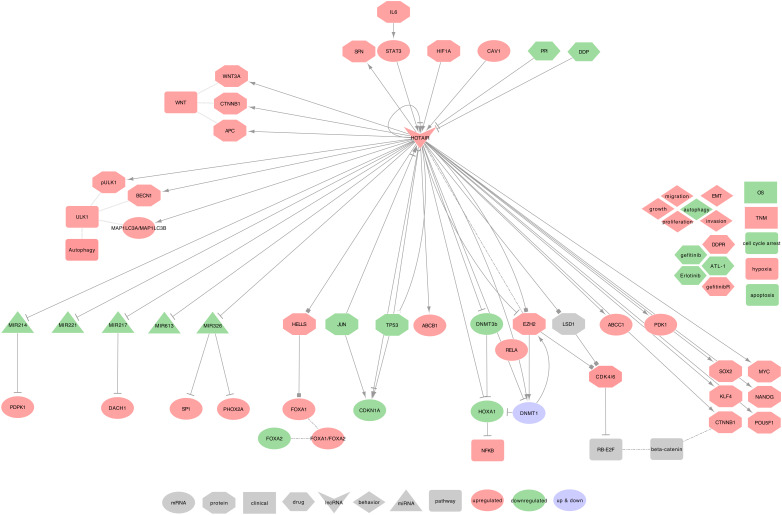
HOTAIR in lung cancers: A network of lncRNA - miRNA - Protein interactions. Upregulated molecules are in green. Downregulated molecules are in red. Up or down-regulated molecules are in purple. Shape definitions are in grey. Gray arrows target the activated molecules. T-ended lines target the inhibited molecules. Square-ended lines represent a binding between molecules. Dashed-dot lines represent an association with unknown interaction.

In Nakagawa et al.’s study on NSCLC tumor tissues, 22.1% of the patients showed at least a two-fold increased expression of HOTAIR. This increase was more frequent in patients with an advanced stage of the tumor than in patients with other stages [98]. Furthermore, HOTAIR expression could upregulate cell migration and anchorage-independent cell growth [87,98,146]. Under hypoxic conditions, HOTAIR also enhances cell proliferation, migration, invasion, EMT, the formation of cancer stem cells (CSCs), and inhibits G0/G1 cell-cycle arrest and cell apoptosis [73,112,216,217,218]. More generally, the HOX cluster-embedded lncRNAs (HOX-lncRNAs) plays a significant role in the regulation of their adjacent coding genes and several HOX-lncRNAs, including HOTTIP, HOXA11-AS, HOTAIRM1, HOXA-AS3, HOXA10-AS, HOTAIR, and HAGLR, which are dysregulated in lung cancer [218]. HOTAIR uses the following mechanisms, as summarized in Figure 11.

⋄ **Regulators of HOTAIR:** In the NSCLC cell line A549, HOTAIR was described as upregulated by hypoxia and CSE [216,217], and is a direct target of HIF-1α (HIF1A), which acts through interaction with putative hypoxia-responsive elements (HREs) in the upstream region of HOTAIR [216]. Besides, pro-inflammatory IL6 can activate STAT3 in an autocrine path, and STAT3 will then increase HOTAIR expression by interacting with its promoter [217]. In NSCLC tumor tissues and A549 cell line, Caveolin 1 (CAV1) was described to upregulate HOTAIR [219]. Interestingly, a specific negative regulation loop involves HOTAIR and TP53 in NSCLC tumor tissues. Indeed, two TP53 binding sites were found on HOTAIR’s promoter and can suppress HOTAIR transcription after TP53 binding. HOTAIR can, in turn, modify the promoter of TP53 by increasing H3K27me3 leading to TP53 repression [220].
⋄ **HOTAIR regulated genes:** In SCLC cell lines (H69 and H446), HOTAIR can activate the NF-κB signaling pathway through the methylation of HOXA1 [73]. Indeed, HOTAIR regulates the HOXA1 methylation level by decreasing DNMT1 and DNMT3B expression [115]. In an NSCLC cell line (PC9), HOTAIR is involved in cellular growth with p65 (RELA), DNMT1, and EZH2. Moreover, HOTAIR can inhibit JUN and CDKN1A [221]. Furthermore, in NSCLC tumors tissues and PC9 cell line, HOTAIR can activate WNT3A, CTNNB1, APC, ABCC1, and ABCB1, and can also promote the expression of 14-3-3σ (SFN) [222,223]. In NSCLC tumor tissues and A459 cell line, HOTAIR would also upregulate CSC-related biomarkers such as NANOG, POU5F1, SOX2, MYC, CTNNB1, and KLF4 [113]. In NSCLC tumor tissues and cell lines (A549, PC9, H1299, and H520), Besides, HOTAIR was associated with LSH (HELLS) to regulate the FOXA1 to FOXA2 ratio and promote cell migration and invasion. Importantly, HELLS regulates this ratio by binding to the promoter of FOXA1, not FOXA2 [224]. Additionally, in Lung cancer cell lines (A549, H460, H1299, NCI-H460, HCC-827), HOTAIR regulates the expression of BECN1, phospho-ULK1, and the LC3II/I (MAP1LC3A / MAP1LC3B) ratio [81].
⋄ **HOTAIR recruits the PRC2:** Fang et al. recently described a negative feedback regulator loop involving HOTAIR in SCLC cell lines (NCI-H69 and NCI-H446). Indeed, HOTAIR may upregulate EZH2 and H3K27me3 levels, which in turn can repress HOTAIR, leading to change HOXA1 methylation [225]. Interestingly, both HOTAIR 5’ and 3’ ends may be involved in the cell cycle dysregulation. Indeed, in NSCLC cell lines (95C, 95D, and YTMLC-90), Liu et al. demonstrated that RB1 and E2F1 are both regulated by HOTAIR5’ via the PRC2 (EZH2, SUZ12, and EED) complex and by HOTAIR3’ via the LSD1/ CoREST/ REST complex. Both complexes may, therefore, act on the WNT/β-catenin signaling pathway and promote EMT when coupled with histone H3 lysine 27 methylation and lysine 4 demethylation [80].
⋄ **HOTAIR as ceRNA:** Among its known functions, HOTAIR was reported as a miRNA regulator. Indeed, HOTAIR can sequester: • hsa-miR-214-3p to release the expression of PDPK1, in NSCLC cell lines (A549 and PC9 cells) [226] • hsa-miR-217 to release the expression of DACH1, in NSCLC cell lines (H23, H292, H1299, and A549) [227] • hsa-miR-326 to release the expression of SP1, in NSCLC tumor tissues and A549 cell line [228], and PHOX2A in NSCLC cell lines (A549, 95D, NCI-H460, HLamp, and H838) [229]. HOTAIR may also sequester hsa-miR-613 in NSCLC tumor tissues and cell lines (H1299, H23, H292, and A549) [230], and hsa-miR-221 in NSCLC tumor tissues and cell lines (A549, H322, and H1299) [100].
⋄ **HOTAIR at the clinical level:** HOTAIR high expression in NSCLC tumor tissues coincides with greater tumor size, advanced TNM stage, lymph node metastasis or lymph-vascular invasion, and short disease-free interval [98]. Its expression was also related to a reduced OS in NSCLC tumor tissues [99]. Furthermore, the expressions of HOTAIR in patients with stage I and II were lower than those with stage III and IV NSCLC tumors [100]. Besides, both H19 and HOTAIR were identified as non-invasive diagnostic biomarkers in the sputum of lung cancer patients [109]. The diagnosis of head-and-neck squamous cell carcinoma (HNSCC) can also be improved by combining the high expression of HOTAIR to the high expression of CASC9 [231]. Altogether, these encouraging results underline the potential of HOTAIR as a diagnostic biomarker.
⋄ **HOTAIR behavior against treatment:** HOTAIR high-expression contributes to DDP resistance via CDKN1A downregulation in LUAD tumor tissues, and experimental downregulated HOTAIR in A549 cells promoted DDP sensitivity [112,113]. It also contributes to Atractylenolide 1 and Erlotinib resistances by activating PDK1 and EZH2, in LUAD cells, in vitro and in vivo [114]. Besides, the downregulation of HOTAIR can increase the SCLC cell lines’ sensitivity to DDP, Adriamycin, and Etoposide, through decreasing DNMT1 and DNMT3B expressions, leading to the reduction of HOXA1 methylation [115]. Furthermore, the downregulation of HOTAIR can increase NSCLC cell lines sensitivity (A549, H460, H1299, NCI-H460, HCC-827) to Crizotinib through the inhibition of ULK1-phosphorylation. This sensitivity leads to the suppression of tumor growth and triggers the cell cycle arrest and the apoptosis signaling pathway [81].
⋄ **Exosomal HOTAIR:** Exosomal HOTAIR (exo-HOTAIR) appeared in the exosomes from bronchoalveolar lavage (BAL) of smokers, NSCLC, and healthy patients, but without significant differences between the three conditions [232]. However, in a recent letter to editors, Zhang et al. found exo-HOTAIR more expressed in the blood samples from LCC patients when compared to LUAD or SCC patients. Moreover, these authors described that A549 and H1299 cells treated with exo-HOTAIR increased the level of cellular HOTAIR. The authors concluded that exo-HOTAIR promotes proliferation, migration, and invasion of the cells through the sequestration of hsa-miR-203 (hsa-mir-203a or hsa-mir-203b) [233]. Interestingly, this interaction between hsa-miR-203 and HOTAIR was also reported with similar effects in renal cell carcinoma cells. In this study, Dasgupta et al. described that the sequestration of hsa-miR-203 decreases CDH1, PTEN, CDKN1A, and CDKN1B levels, while it increases the expression of VIM [234].

### 2.8. GAS5

Growth Arrest Specific 5 (GAS5) is an RNA gene localized on the cytogenetic band 1q25.1. GAS5 gene has 31 transcripts, 20 identified as retained introns, and 11 identified as lncRNAs. GAS5 can bind the DNA binding domain of the glucocorticoid receptor, which disable it from regulating the transcription of its target genes.

#### 2.8.1. GAS5 and Asthma

In Qiu et al. study, the heatmap Figure 2A displays an upregulated GAS5 in CD4${}^{+}$T-cells from patients with severe asthma vs. healthy patients, with a fold change greater than 2 [59]. These results add to Keenan et al. previous observations in bronchial epithelial cells (BEAS-2B) and primary human airway smooth muscle (ASM) cultures. Indeed, pro-inflammatory mediators, TNF, and IL1α (IL1A) were observed to promote GAS5 upregulation in ASM and BEAS-2B cells, which in turn can modulate glucocorticoid activity and thus may mediate glucocorticoid insensitivity [60].

#### 2.8.2. GAS5 and Lung Cancer

**Figure 12:**
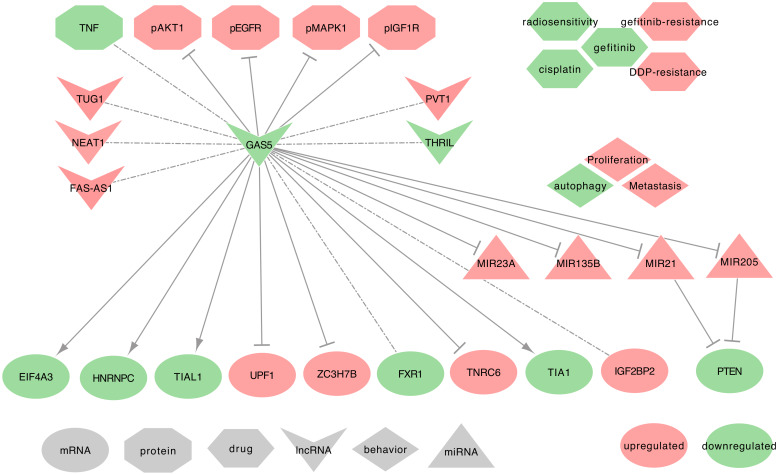
GAS5 in lung cancers: A network of lncRNA - miRNA - Protein interactions. Upregulated molecules are in green. Downregulated molecules are in red. Shape definitions are in grey. Gray arrows target the activated molecules. T-ended lines target the inhibited molecules. Square-ended lines represent a binding between molecules. Dashed-dot lines represent an association with unknown interaction.

GAS5 expression levels are low in lung cancers. Interestingly, this decrease in NSCLC is significant in male donors only [95]. GAS5 is involved in cellular proliferation, metastasis, and autophagy. GAS5 uses the following mechanisms, as summarized in Figure 12.

⋄ **GAS5 regulated genes:** In NSCLC tumor tissues, GAS5 decrease correlates strongly and significantly to the decrease of FAS-AS1 and THRIL in male donors and combined tumors, as well as the increase of NEAT1 in male donors and combined tumors. GAS5 decrease also correlates with TUG1 increase in female donors and combined tumors, and with PVT1 increase in female donors when compared to ANCTs. GAS5 expression is also associated with the upregulation of IGF2BP2 and the downregulation of FXR1 [95]. Moreover, GAS5 downregulates the expression of TNRC6A, ZC3H7B, and UPF1, while it can upregulate the expression of EIF4A3, TIA1, TIAL1, and HNRNPC [95]. Besides, in NSCLC tumor tissues and cell lines (A549, H1299, H1975, HCC827), GAS5 can deregulate the expression of phospho-EGFR, phospho-MAPK1, phospho-AKT1, and IGF1R. Interestingly, GAS5 overexpression inversely correlates with the activation of the EGFR pathway [110].
⋄ **GAS5 as ceRNA:** Among its known functions, GAS5 can act as a miRNA regulator. Indeed, GAS5 may sequester hsa-miR-21-5p in NSCLC tumor tissues and the cell lines (NCI-H460, A549, NCI-H1299, H460, SK-MES-1, H157, and H358) [82,111]. GAS5 may also sequester hsa-miR-205-5p in the NSCLC cell lines (A549, H460, 95D, H1299, SPC-A-1, and H522) [235]. Both hsa-miR-21-5p and hsa-miR-205-5p sequestration would release the expression of PTEN [82,111,235]. Additionally, GAS5 suspected of sequestering hsa-miR-135b-5p in NSCLC tumor tissues and cell lines (A549 and H1975) [236]. It is also suspected to sequester hsa-miR-23a in NSCLC tumor tissues and cell lines (A549, H838, H157, and HCC827) [237].
⋄ **GAS5 at the clinical level:** Esfandi et al. emphasized the GAS5 low expression in tumor tissues as a promising biomarker for the diagnosis of the NSCLCs [95].
⋄ **GAS5 behavior against treatment:** GAS5 may regulate chemo-resistance to DDP of NSCLC tumor tissues and cell lines (H460 and H157), through the PTEN signaling pathway [82]. Besides, GAS5 low expression contributes to resistance to gefitinib to LUAD cell lines and tumor tissues [110]. Furthermore, its low expression can promote the resistance to ionizing radiation in NSCLC cell lines [111] and tumor tissues [236].
⋄ **Exosomal GAS5:** In Cheng et al. study on urethane-induced lung cancer mouse model, lung cancer-derived exosomal GAS5 (exo-GAS5) affects the proliferation, apoptosis, and tube formation of human umbilical vein endothelial cells (HUVECs). The overexpression of GAS5 leads to an increase of exo-GAS5, upregulates PTEN expression, and inhibits the phosphorylation of PI3K/AKT, through hsa-miR-29b-3p sequestration [238]. Furthermore, exo-GAS5 expression was lower in the serum of 64 NSCLC patients when compared to healthy controls. This low expression of exo-GAS5 was associated with larger tumor size and advanced TNM [239]. While being a potential new biomarker for the diagnosis of Stage I NSCLCs, further studies should be conducted on larger cohorts to confirm the predictive power of exo-GAS5.

## 3. Additional LncRNAs Not Yet Described in Lung Cancer Studies

### 3.1. LINC00861

Long Intergenic Non-Protein Coding RNA 861 (LINC00861) is an RNA gene localized on the cytogenetic band 8q24.13. LINC00861 gene has 9 transcripts, all identified as lncRNAs.

#### 3.1.1. LINC00861 in Asthma

In RNAseq available data, LINC00861 transcript 201 (LINC00861-201) was found upregulated in eosinophilic asthma and eosinophilic asthma with high Igg patients vs. healthy patients [61].

#### 3.1.2. LINC00861 in COPD

In COPD smokers, LINC00861 expression is lower than in non-smoking COPD or healthy patients. LINC00861 and the uncharacterized LOC101928100 RNA gene were co-expressed with RORA, while the authors observed an upregulation of hsa-miR-218-5p. The authors hypothesized that the LINC00861/LOC101928100 upregulation releases the expression of RORA through the sequestration of both hsa-miR-218-5p and hsa-miR-15a [240].

### 3.2. CCDC18-AS1, RP4-717I23.3

CCDC18-AS1 is an RNA gene localized on the cytogenetic band 1p22.1. CCDC18-AS1 gene has 32 transcripts, all identified as lncRNAs.

#### 3.2.1. CCDC18-AS1 in Asthma

In asthma and severe asthma when compared to healthy controls, CCDC18-AS1 was upregulated, as shown by Figure 2B of Chen et al. study [241].

#### 3.2.2. CCDC18-AS1 in COPD

In PBMCs of COPD smokers, CCDC18-AS1 transcripts (CCDC18-AS1-220 and CCDC18-AS1-214) are upregulated when compared with COPD nonsmokers [242].

## 4. Additional Interesting Exosomal-LncRNAs Described with Lung Cancer

### 4.1. Exo-UCA1

Urothelial Cancer-Associated 1 (UCA1) is an RNA gene localized on the cytogenetic band 19p13.12. UCA1 has 45 known transcripts, all of them described as lncRNAs. UCA1 can promote cell proliferation and resistance to gefitinib-induced cell apoptosis. It can also sequester miR-143 to release FOSL2 expression, leading to gefitinib resistance of epidermal growth factor receptor-positive (EGFR+) NSCLCs [243]. 

**exo-UCA1 behavior against in lung cancer treatment** Kwok et al. demonstrated that the transfer of extracellular Vesicle-Associated-RNAs could induce drug resistance in ALK-Translocated lung Adenocarcinoma [244]. Moreover, exo-UCA1 levels are high in both gefitinib-resistant NSCLC cells and their secreted exosomes [243].

### 4.2. Exo-lncMMP2-2

Wu et al. recently described exosomal lnc-MMP2-2-1_dup1 could regulate migration and invasion of lung cancer cells to the vasculature by promoting MMP2 expression [245].

### 4.3. Exo-GAPLINC

Gastric Adenocarcinoma Associated, Positive CD44 Regulator, Long Intergenic Non-Coding RNA (GAPLINC) is an RNA gene localized on the cytogenetic band 18p11.31. GAPLINC has four transcripts, all described as lncRNAs. Exosomal GAPLINC (exo-GAPLINC), was described to promote erlotinib resistance in NSCLCs [246]. Erlotinib is a tyrosine kinase inhibitor that is effective in patients with or without EGFR mutations but appears to be more effective in patients with EGFR mutations.

### 4.4. Exo-TBILA and Exo-AGAP2-AS1

TGF-Beta Induced lncRNA (TBILA) is an RNA gene localized on the cytogenetic band 3q13.2. AGAP2 Antisense RNA 1 (AGAP2-AS1) is an RNA gene localized on the cytogenetic band 12q14.1. Both TBILA and AGAP2-AS1 have a unique transcript identified as a lncRNA. Both exosomal TBILA (exo-TBILA) and exosomal AGAP2-AS1 (exo-AGAP2-AS1) expression are higher in the serum of NSCLC patients than in exosomes-depleted serum (EDS) from NSCLC patients or serum of healthy patients. Importantly, these two lncRNAs are very stable in the bloodstream, which makes them promising biomarkers. Moreover, they have only one known transcript each, which reduces potential screening errors. TBILA can discriminate all NSCLC patients, while AGAP2-AS1 is better as distinguishing SCC patients from healthy controls. Additionally, the authors mentioned that the combination of TBILA/AGAP2-AS1 with Cyfra21 (KRT19), a protein widely used in clinical practices, could distinguish all NSCLC patients from healthy controls in their study [247].

### 4.5. Exo-SOX2-OT

SOX2 Overlapping Transcript (SOX2-OT) is an RNA gene localized on the cytogenetic band 3q26.33. SOX2-OT has 104 known transcripts, all identified as lncRNAs Exosomal SOX2-OT (exo-SOX2-OT) was significantly upregulated in Lung Squamous Cell Carcinoma (LSCC) patients when compared to non-LSCC patients. Exo-SOX2-OT levels in plasma correlated with tumor size and TNM stages, and might also reflect SOX2-OT expression in tumors [248]. Nevertheless, with 104 known transcripts, all identified as lncRNAs, further studies should identify which transcripts are useful for diagnosis.

## 5. Concluding Remarks and Future Perspectives

LncRNAs are promising molecules for the better understanding of protein-gene regulations and subsequent pathways that define lung diseases. In the current review, we attempted to cover well-described lncRNAs associated with at least two lung diseases within asthma, IPF, COPD, and lung cancers. First, we searched into the PubMed database for publications related to lncRNAs in each of the four lung diseases. Secondly, we built and described the networks of molecular interactions of the lncRNAs H19, MALAT1, MEG3, FENDRR, CDKN2B-AS1, TUG1, HOTAIR, and GAS5, within each disease. Thirdly, we reported the clinical relevance of each of these lncRNAs, focusing on the biomarker and the treatment response aspects. Finally, we covered ten additional lncRNAs that were described only in lung cancers under their exosomal form.

Based on the literature covered here, it is evident that the interaction networks are far more complex than those presented here. Indeed, these networks are an extension of the following signaling pathways found in common for different diseases or lncRNAs: • The WNT/β-catenin signaling pathway, has been associated with H19, MALAT1, and HOTAIR in NSCLC [74,75,76,80] • FENDRR and H19 are upstream regulators of the fibrosis and associated with the TGFB/SMAD3 signaling pathway in IPF [64,65] • MEG3 and HOTAIR target the apoptosis pathway in COPD and NSCLC, respectively [70,81]. • GAS5, TUG1, H19, and MALAT1 are upstream regulators of the PTEN/PI3K/AKT signaling pathway in NSCLC [77,78,82,83,111,135,235] • ANRIL (CDKN2B-AS1) is an upstream regulator of the P53 signaling pathway in both IPF and NSCLC [63]. Hence, all these studies on lncRNAs increased the list of upstream regulators of crucial cancer signaling pathways.

These lncRNAs have, therefore, the potential to sustain the tumor state of a cell. They were otherwise previously described as oncogenes and tumor-suppressors in lung cancers. In this context, H19, HOTAIR, MALAT1, and CDKN2B-AS1 are oncogenic lncRNAs [87,176,249,250,251], while MEG3, FENDRR, and GAS5 are tumor suppressor lncRNAs [116,154,252,253,254,255]. Intriguingly, TUG1 can be one or the other regarding lung cancer subtype. TUG1 would, therefore, act as an oncogene in SCLCs and many human cancers, but would act as a tumor-suppressor in NSCLCs [256]. Therefore, such versatile behavior suggests either a tumor-specific mechanism of action or the presence of an upstream regulator with a tumor-dependent expression.

Moreover, we can link these interaction networks to the hallmarks of cancer. Due to their implication in multiple steps of the cancer progression, we can group the eight lncRNAs according to Hanahan and Weinberg’s classification [257] under the following hallmarks:

⋄ *Genome instability and mutation:* MALAT1 and CDKN2B-AS1 may be key players of the “Genome instability and mutation” hallmark since they can decrease the expression levels of PARP1 [87,121,171]. H19 may also contribute to the “Genome instability and mutation” as well as the “Evading growth suppressors” hallmarks. CSC exposure induces an overall increase of H3K27me3 levels, which would repress many genes [74]. However, RIOX2 may demethylate H19 before the DNA-repair gene MGMT and the cyclin-dependent kinase inhibitor p16-CDKN2A are methylated [74,139]. Subsequently, a decreased level of MGMT would lead to chromosomal alterations, while a decrease in p16-CDKN2A would lead to inhibit the cell cycle arrest in G1 and G2 phases.
⋄ *Activating invasion and metastasis:* FENDRR may be a key player of the “Activating invasion and metastasis” hallmark. The low FENDRR expression observed in NSCLC tumor tissues allows the ECM degradation by the metalloproteinases and thus facilitates the metastasis. Indeed, under normal conditions, FENDRR is supposed to sequester hsa-miR-761, which will permit the increase of the metalloproteinase inhibitor TIMP2, leading to the degradation of the extracellular matrix [196].
⋄ *Resisting cell death:* MALAT1, MEG3, and CDKN2B-AS1 may be key actors of the “Resisting cell death” hallmark. CDKN2B-AS1 decreases cleaved-CASP3 while increasing BCL2 and CASP3 expression [87,121]. High levels of MEG3 reduces CASP3 through hsa-miR-205-5 sequestration [154,157,160]. These high levels can also decrease the expression of BIRC5 [118]. MALAT1 can also decrease cleaved-CASP3 levels [171]. Subsequently, in NSCLCs, cleaved-CASP3 is decreased, while BCL2, BIRC5, and CASP3 is increased, which leads to escape apoptosis.
⋄ *Sustaining and proliferative signaling:* GAS5, TUG1, MALAT1, H19, and HOTAIR may be key actors of the “Sustaining and proliferative signaling” hallmark in NSCLCs. High GAS5 and low HOTAIR levels combined with EGFR inhibitors, increase the sensitivity to treatment [110,114]. GAS5, TUG1, H19, and MALAT1 are upstream regulators of the PTEN/PI3K/AKT signaling pathway. H19 recruits EZH2 to repress PTEN expression [135]. MALAT1 is involved in the upregulation of PIK3CA [77] and phospho-STAT3 [171], and in the phosphorylation of AKT1 and MTOR [79,102]. Finally, TUG1 and GAS5 can release PTEN expression, respectively, through hsa-miR-221 and hsa-miR-21-5p, hsa-miR-205-5p sequestration [82,83,111,235]. Subsequently, in NSCLCs, PTEN is downregulated, while PIK3CA, phospho-STAT3, phospho-AKT1, and phospho-MTOR are increased, thus enhancing the cellular proliferation.

The eight lncRNAs may provide an interesting angle to circumvent the treatment resistances observed in lung cancers. Throughout the review, we listed treatment resistance to Cisplatin, Vincristine, Paclitaxel, Erlotinib, Atractylenolide 1, Adriamycin, Etoposide, Crizotinib, Gefitinib, and ionizing radiations. Surprisingly, HOTAIR alone was involved in the resistance to Crizotinib, Cisplatin, Erlotinib, and Atractylenolide 1 in NSCLC cells [81,112,113,114], and to Cisplatin, Adriamycin, and Etoposide in SCLC cells [115]. Although these results require confirmation, HOTAIR could be a promising target for future treatments. These results also underline the importance of studying lncRNAs in lung cancer.

The lncRNAs described here may be promising non-invasive biomarkers for the diagnosis or prognosis of asthma, COPD, and lung cancers. Indeed, GAS5, MEG3, MALAT1, or CDKN2B-AS1 may be used to diagnose asthma [58,59,61]. TUG1 could be used to diagnose the COPD, and in association with CDKN2B-AS1, it could also help predict acute exacerbations in COPD patients [67,71]. In IPF patients, CDKN2B-AS1 is again a new biomarker that could predict the occurrence of lung cancers [63]. In lung cancers, MALAT1 and H19 are interesting for the diagnosis of NSCLCs [89,90,91,92,93]. TUG1 is also a promising biomarker for diagnosis of LUAD [86], whereas H19 or HOTAIR can help discriminate SCC from LUAD [109]. Nevertheless, while being very encouraging results, most of these studies lack some confirmation from additional studies in large independent cohorts. Furthermore, the majority of these lncRNAs studied as individual biomarkers may help define better the classification of lung diseases. For example, using machine learning algorithms on previously established biomarkers combined with these lncRNAs, new disease subtypes may be revealed.

Accumulative pieces of evidence display lncRNAs as communication entities to regulate their micro-environment, thus being potentially involved in a local disease spread. While non-cancerous diseases have received some attention, such as osteoarthritis or chronic inflammatory diseases including rheumatoid arthritis, systemic lupus erythematosus, and psoriasis [258], we mentioned the lack of studies covering exosomal-lncRNAs in asthma, COPD, and IPF. Hence, further efforts are needed to identify exosomal-lncRNAs in these three lung diseases. Conversely, the studies on exosomal-lncRNAs in lung focused on cancers. Although we did not find any shreds of evidence that exosomes transport MEG3, FENDRR, TUG1, and CDKN2B-AS1 in lung cancers, the mechanisms of action of these lncRNAs strongly suggest their involvement in cancer invasion. First, these lncRNAs may trigger important hallmarks of cancers, such as “Genome instability and mutation” for CDKN2B-AS1 [87,121], “Activating invasion and metastasis” for FENDRR [196], “Resisting cell death” for MEG3 and CDKN2B-AS1 [118,154,157,160,207], and “Sustaining and proliferative signaling” for TUG1 [83]. Secondly, previous studies in other cancers found an exosomal expression of MEG3, TUG1, and CDKN2B-AS1 [163,164,165,201,202,215]. These findings suggest that MEG3, TUG1, and CDKN2B-AS1 may also be involved in lung cancer intercellular communication. Surprisingly, FENDRR remains unassociated with exosomes in any diseases. Nevertheless, its implication in invasion and metastasis [196] suggests a late expression during the tumor progression, which could explain the lack of literature.

Altogether, throughout these numerous studies, we first underlined the importance of describing the lncRNAs mechanisms of action. By continuously seeking their putative regulators and downstream targets, new diagnostic tools and further treatments may arise. This review also compiled knowledge on exosomal-lncRNAs in lung diseases, and emphasize the essential position of some lncRNAs in the transcription regulation. Hence, we would be thrilled to read more investigations, in lung cancer, on exosomal MEG3, FENDRR, TUG1, and CDKN2B-AS1 or treatment resistance involving HOTAIR. We would be even more thrilled to read investigations on exosomal-lncRNAs in asthma, COPD, and IPF. Here, we highlighted eight well-described members of the lncRNAs’ ocean. While these lncRNAs may trigger new research interests, it is of prime importance to identify all of them and to describe their mechanisms of action to better understand lung diseases.

## Figures and Tables

**Table 1:** Downstream targets of the 8 lncRNAs described in lung diseases.

Disease	LncRNA	Expression	Location	Action	Targeted Pathway	Downstream Targets	References
Asthma	CDKN2B-AS1	up	BA-E & BA-R	Pro-inflammatory	-	TNF, IL17A	[58]
	GAS5	up	Severe asthma CD4${}^{+}$T-cells	Treg/Th17 balance	-	FOXP3, RORC	[59]
			BEAS-2B and primary human ASM cell cultures	Glucocorticoid activity	-	-	[60]
	MALAT1	up	blood of highly-expressed IgE eosinophilic asthma	Inhibits pathway	T-cell receptor	-	[61]
			Severe asthma CD4${}^{+}$T-cells	Treg/Th17 balance	-	FOXP3, RORC	[59]
	MEG3	up	Severe asthma CD4${}^{+}$T-cells	Pro-inflammatory	Th17 cell differentiation	IL17A, IL22, RORC	[59]
	TUG1	up	ASM of Sprague Dawley rats	Promotes cell proliferation and migration	-	Fgf1	[62]
IPF	CDKN2B-AS1	down	peripheral blood	Activates cell cycle arrest	P53	CDKN2A, TP53	[63]
	FENDRR	down	fibrotic human lung cells and mouse primary lung fibroblasts	Inhibits fibroblast activation & reduces pulmonary fibrosis	TGFB / SMAD3	ACO1	[64]
	H19	up	human pulmonary fibrotic tissues	Induces fibrosis	TGFB / SMAD3	TGB1, SMAD3	[65]
	MEG3	up	pulmonary epithelial cells from IPF lung tissue	Promotes cell migration	-	TP63, KRT14, STAT3, YAP1, TP73, SOX2, HES1, HEY1	[66]
COPD	CDKN2B-AS1	down	plasma of AECOPD	Anti-inflammatory	-	TNF, IL1B, IL17A, CXCL8	[67]
	H19	up	Quadriceps of FFMI patients with COPD	Susceptibility to low FFMI	-	MYOD1	[68]
	HOTAIR	up	CS-exposed male BALB/c mice & HBE cells treated with CSE	-	-	IL6, CXCL8, CDH2, VIM, ACTA2, CDH1	[69]
	MEG3	up	lung from COPD & CSE-treated 16HBE cells	Induces apoptosis and inflammation	Apoptosis	IL1B, IL6, TNF	[70]
	TUG1	up	sputum and lung from COPD smokers & non-smokers	Inhibits inflammation and airway remodelling	-	DUSP6	[71]
			TGFB1 treated BEAS-2B and HFL1 cells	Inhibits cell proliferation	-	ACTA2, FN1	[72]
SCLC	HOTAIR	up	H69 and H446 cell lines	Activates the pathway	NF-κB	HOXA1	[73]
NSCLC	H19	up	CDK-4/hTERT-immortalized HBEC	Associated with pathway activation	WNT/β-catenin	WNT2, WNT5A, WNT6, WNT10A, FOXN1, TCF7	[74]
	MALAT1	up	Tumor tissues and H1299 cell line	Associated with pathway activation	WNT/β-catenin	-	[75,76]
			H1299 and H520 cell lines	Activates the pathway	PTEN / PI3K / AKT	BCL2, MMP9, PIK3CA, STAT3	[77,78]
			Tumor tissues & A549 and H1299 cell lines	Regulates the pathway	AKT / MTOR	-	[79]
	CDKN2B-AS1	down	Peripheral blood of IPF	Regulates the pathway	P53	-	[63]
	HOTAIR	up	95C, 95D and YTMLC-90 cell lines	Regulates the pathway	WNT/β-catenin	RB1, E2F1	[80]
			A549, H460, H1299, NCI-H460 and HCC-827 cell lines	Activates the pathway	Apoptosis	pULK1	[81]
	GAS5	down	Tumor tissues & A549, NCI-H1299, H460, SK-MES-1, H157, and H358 cell lines	Regulates the pathway	PTEN / PI3K / AKT	PTEN	[82]
	TUG1	down	Tumor tissues & SPC-A1, NCI-H520, NCI-H520 and NCI-H1299 cell lines	Regulates the pathway	PTEN / PI3K / AKT	PTEN	[83]

**Table 2:** Potential biomarkers from the eight lncRNAs observed in lung diseases.

Disease	Location	LncRNA	Type	Value	Relevancy	References
Asthma	CD4${}^{+}$T-cells	GAS5 / MEG3	expression	upregulated	Up in asthmatic patients vs. healthy patients	[59]
	peripheral whole blood	MALAT1	expression	upregulated	Up in highly-expressed IgE eosinophilic asthmatic (EA) patients vs. healthy patients	[61]
	plasma	CDKN2B-AS1	expression	upregulated	Up in patients with bronchial asthma vs. healthy patients	[58]
COPD	plasma	CDKN2B-AS1	expression	downregulated	Down in patients with acute exacerbations of COPD vs. stable COPD or healthy patients	[67]
	sputum & lung	TUG1	expression	upregulated	Up in COPD patients with or without smoking history	[71]
IPF	peripheral whole blood	CDKN2B-AS1	expression	downregulated	Down in IPF patients, vs. healthy controls May promote the occurrence of lung cancers	[63]
Lung cancers	constitutive DNA	H19	polymorphism	rs217727 C >T	Associated with increased risk of lung cancer in meta-analysis	[84]
		MEG3			rs4081134 G >A	Genotype [AA] associated with lung cancer risk in chinese northeast population	[85]
LUAD	serum	TUG1	expression	upregulated	Up in LUAD patients vs. healthy patients	[86]
	tumor	CDKN2B-AS1	expression	upregulated	Up in cell lines & positively correlated with the differentiation grade and the TNM stages	[87]
	tumor	FENDRR	expression	downregulated	Strongly associated with High TNM 1 stage in LUAD patients vs. healthy patients. Predicts LUAD cancer vs. healthy state when associated with LINC00312	[88]
NSCLC	plasma	H19	expression	upregulated	Up in NSCLC vs. begnin lung disease	[89]
	serum	MALAT1	expression	downregulated	Down in patients with NSCLC vs. healthy patients	[90,91,92,93]
	tumor	CDKN2B-AS1	expression	upregulated	Correlated with poor patient OS	[94]
		GAS5	expression	downregulated	Down in male subjects vs. corresponding ANCTs.	[95]
		H19	expression	upregulated	Up in stage III and IV vs. stage I and II & negatively correlated with OS	[96,97]
		HOTAIR	expression	upregulated	Up in patients with stage III and IV vs. stage I and II. Positively correlated with greater tumor size, lymph node metastasis or lymph-vascular invasion, short disease free interval, and reduced OS.	[98,99,100]
		MALAT1	expression	upregulated	Associated with a poor prognosis and short OS. Associated with age, tumour size & TNM stage, when combined to SOX9	[76,79,101,102,103,104,105]
		MEG3	expression	downregulated	Associated with short-term survival	[106]
		TUG1	expression	downregulated	Associated with a high TNM stage and a poor patient outcome	[105,107,108]
SCC	sputum	H19/HOTAIR	expression	-	Diagnosis of SCC vs. LUAD	[109]

**Table 3:** Lung cancer treatment resistances associated to the eight lncRNAs described in lung diseases.

Disease	Treatment	LncRNA	Expression	Relevancy	References
LUAD	Gefitinib	GAS5	down	Overexpression increases sensitivity to treatment	[110]
	Ionizing radiation			Overexpression increases radiosensitivity	[111]
	Cisplatine	HOTAIR	up	Repression increases sensitivity to treatment	[112,113]
	Erlotinib			Repression of PDK1 and HOTAIR-mediated EZH2 gene expression increases sensitivity to treatment	[114]
	Atractylenolide 1				
SCLC	Cisplatine	HOTAIR	up	Repression increases sensitivity to treatment	[115]
	Adriamycin				
	Etoposide				
NSCLC	Cisplatine	FENDRR	down	Negatively correlated with treatment response	[116]
		GAS5	down	Could regulate chemo-resistance to treatment	[82]
		H19	up	Negatively correlated with treatment response	[96]
		MALAT1	up	Increases resistance to treatment through positive feedback loop with SOX9	[76]
		MEG3	down	Overexpression increases sensitivity to treatment	[117,118,119]
		TUG1	down	Overexpression increases sensitivity to treatment	[83]
	Crizotinib	HOTAIR	up	Repression increases sensitivity to treatment	[81]
	Paclitaxel	CDKN2B-AS1	up	Inhibits sensitivity to treatment	[87]
	Vincristine	MEG3	down	Overexpression increases sensitivity to treatment	[117,120]
OSCC	Cisplatine	CDKN2B-AS1	up	Associated with Midkine to treatment resistance	[121]

## Abbreviations

The following abbreviations are used in this manuscript:

**General**

**Abbreviation**	**Full name**
AECOPD	acute exacerbations of COPD
ANCT	Adjacent Non-Cancerous Tissue
ASM	Airway Smooth Muscle
BA-E	Bronchial asthma at exacerbation
BA-R	Bronchial asthma at remission
BEAS-2B	Bronchial epithelial cells
ceRNA	competing endogenous RNA
CDK	Cyclin Dependent Kinase
COPD	Chronic Obstructive Pulmonary Disease
CS	Cigarette Smoke
CSC	Cancer Stem Cell
CSE	Cigarette Smoke Extract
DDP	Cisplatine
EMT	Epithelial Mesenchymal Transition
FEV_1	Forced expiratory volume in 1 second
FFMI	Fat-free mass index
FVC	Forced vital capacity
GINA	Global Initiative for Asthma
H3K9me3	H3 lysine 9 tri-methylation
H3K27me3	H3 lysine 27 tri-methylation
HBE	Human Bronchial Epithelial cells
HRE	Hypoxia-Responsive Element
HNSCC	head-and-neck squamous cell carcinoma
IPF	Idiopathic Pulmonary Fibrosis
LD	linear dichroism
LDL	low-density lipoprotein
LSCC	Lung Squamous Cell Carcinoma
lncRNA	long non-coding RNA
LTB4	Leukotriene B4
LUAD	Lung Adenocarcinoma
MET	Mesenchymal Epithelial Transition
NAT	Natural Antisense transcripts
NSCLC	Non Small Cell Lung Cancer
OS	Overall Survival
OSCC	Oral Squamous Cell Carcinoma
PRC	Polycomb Repressive Complex
PTX	Paclitaxel
SCC	Squamous Cell Carcinoma
SCLC	Small Cell Lung Cancer
SNP	Single Nucleotide polymorphism
TCGA	The Cancer Genome Atlas
TNM	Tumor-Node–Metastasis:
TNM	T: Extent of the primary tumor,
TNM	N: lymph node involvement,
TNM	M: metastatic disease
WHO	World Health Organization

**Signaling Pathway**

**Signaling Pathway**	**KEGG ID**
AKT/MTOR signaling pathway	hsa04150
Apoptosis signaling pathway	hsa04210
EGFR pathway	hsa01521
NF-κB signaling pathway	hsa04064
PTEN/PI3K/AKT signaling pathway	hsa04151
TGFB/SMAD3 signaling pathway	hsa04350
T-cell receptor signaling pathway	hsa04660
Th17 cell differentiation	hsa04659
P53 signaling pathway	hsa04115
WNT/β-catenin signaling pathway	hsa04310

**miRNAs**

**Mirbase ID**	**HGNC symbol**	**Mirbase accession**
hsa-miR-101-3p	MIR101-1	MIMAT0000099
hsa-miR-106a	MIR106A	MI0000113
hsa-miR-106b	MIR106B	MI0000734
hsa-miR-107	MIR107	MI0000114
hsa-miR-124-1	MIR124-1	MI0000443
hsa-miR-125a	MIR125A	MI0000469
hsa-miR-135b-5p	MIR135B	MIMAT0000758
hsa-miR-140	MIR140	MI0000456
hsa-miR-142-3p	MIR142	MIMAT0000434
hsa-miR-145	MIR145	MI0000461
hsa-miR-145-5p	MIR145	MIMAT0000437
hsa-miR-148a	MIR148A	MI0000253
hsa-miR-15a	MIR15A	MI0000069
hsa-miR-195	MIR195	MI0000489
hsa-miR-196b	MIR196B	MI0001150
hsa-miR-197-3p	MIR197	MIMAT0000227
hsa-miR-17	MIR17	MI0000071
hsa-miR-196a-1	MIR196A1	MI0000238
hsa-miR-196a-2	MIR196A2	MI0000279
hsa-miR-200	MIR200 family	hsa-miR-200 family
hsa-miR-200a	MIR200A	MI0000737
hsa-miR-200a-3p	MIR200A	MIMAT0000682
hsa-miR-200b	MIR200B	MI0000342
hsa-miR-200c	MIR200C	MI0000650
hsa-miR-203a	MIR203A	MI0000283
hsa-miR-203b	MIR203B	MI0017343
hsa-miR-204	MIR204	MI0000284
hsa-miR-205-5p	MIR205	MIMAT0000266
hsa-miR-206	MIR206	MI0000490
hsa-miR-21	MIR21	MI0000077
hsa-miR-21-5p	MIR21	MIMAT0000076
hsa-miR-210	MIR210	MI0000286
hsa-miR-214	MIR214	MI0000290
hsa-miR-214-3p	MIR214	MIMAT0000271
hsa-miR-217	MIR217	MI0000293
hsa-miR-218-1	MIR218-1	MI0000294
hsa-miR-218-5p	MIR218-1	MIMAT0000275
hsa-miR-218-2	MIR218-2	MI0000295
hsa-miR-221	MIR221	MI0000298
hsa-miR-23a	MIR23A	MI0000079
hsa-miR-29b-3p	MIR29B1	MIMAT0000100
hsa-miR-301b	MIR301B	MI0005568
hsa-miR-3163	MIR3163	MI0014193
hsa-miR-326	MIR326	MI0000808
hsa-miR-33a-5p	MIR33A	MIMAT0000091
hsa-miR-409-3p	MIR409	MIMAT0001639
hsa-miR-429	MIR429	MI0001641
hsa-miR-484	MIR484	MI0002468
hsa-miR-519a-1	MIR519A1	MI0003178
hsa-miR-519a-2	MIR519A2	MI0003182
hsa-miR-590-5p	MIR590	MIMAT0003258
hsa-miR-613	MIR613	MI0003626
hsa-miR-650	MIR650	MI0003665
hsa-miR-675	MIR675	MI0005416
hsa-miR-675-5p	MIR675	MIMAT0004284
hsa-miR-6515-3p	MIR6515	MIMAT0025487
hsa-miR-7-5p	MIR7-1	MIMAT0000252
hsa-miR-761	MIR761	MI0003941
hsa-let-7	MIRLET7 family	hsa-let-7 family
hsa-let-7d-5p	MIRLET7D	MIMAT0000065

**lncRNAs**

**HGNC symbol**	**Alias**	**Full name**	**Type**	**Accession ID**
AGAP2-AS1		AGAP2 Antisense RNA 1	NAT	ENSG00000255737
CCDC18-AS1		CCDC18 Antisense RNA 1	NAT	ENSG00000223745
CDKN2B-AS1	ANRIL	CDKN2B Antisense RNA 1	NAT	ENSG00000240498
FAS-AS1		FAS Antisense RNA 1	NAT	HGNC:37128
FENDRR		FOXF1 adjacent non-coding		
		developmental regulatory RNA	NAT	ENSG00000268388
GAPLINC	RP11-838N2.4	Gastric adenocarcinoma associated,		
		positive CD44 regulator, lincRNA	lincRNA	ENSG00000266835
GAS5		Growth Arrest Specific 5	NAT	ENSG00000234741
H19		H19 Imprinted Maternally Expressed Transcript	NAT,	ENSG00000130600
			lincRNA	
HAGLR		HOXD Antisense Growth-Associated lncRNA	NAT	ENSG00000224189
HOTAIR		HOX Transcript Antisense RNA	NAT	ENSG00000228630
HOTAIRM1		HOXA Transcript Antisense RNA,		
		Myeloid-Specific 1	NAT	ENSG00000233429
HOTTIP		HOXA Distal Transcript Antisense RNA	NAT	ENSG00000243766
HOXA-AS3		HOXA Cluster Antisense RNA 3	NAT	ENSG00000254369
HOXA10-AS		HOXA10 Antisense RNA	NAT	ENSG00000253187
HOXA11-AS		HOXA11 Antisense RNA	NAT	ENSG00000240990
LINC00312		Long Intergenic Non-Protein Coding RNA 312	lincRNA	HGNC:6662
LINC00861		Long Intergenic Non-Protein Coding RNA 861	lincRNA	ENSG00000245164
-	lnc-MMP2-2	lnc-MMP2-2-1_dup1	lincRNA	NONHSAT142627
MEG3		Maternally Expressed 3	NAT	ENSG00000214548
MALAT1		Metastasis Associated LUAD Transcript 1	NAT	ENSG00000251562
NEAT1		Nuclear Paraspeckle Assembly Transcript 1	lincRNA	ENSG00000245532
PVT1		Pvt1 Oncogene	NAT	ENSG00000249859
THRIL		TNF And HNRNPL Related		
		Immunoregulatory lncRNA	lincRNA	ENSG00000280634
TUG1		Taurine Up-Regulated 1	lincRNA	ENSG00000253352
TBILA		TGF-Beta Induced LncRNA	lincRNA	ENSG00000261488
SOX2-OT		SOX2 Overlapping Transcript	NAT	ENSG00000242808
UCA1		Urothelial Cancer Associated 1	lincRNA	ENSG00000214049
ZEB1-AS1		ZEB1 Antisense RNA 1	NAT	ENSG00000237036

**Genes**

**HGNC symbol**	**Alias**	**Full name**	**Accession ID**
ABCB1	MDR1	ATP Binding Cassette Subfamily B Member 1	ENSG00000085563
ABCC1	MRP1	ATP Binding Cassette Subfamily C Member 1	ENSG00000103222
ABCC10		ATP binding cassette subfamily C member 10	ENSG00000124574
ACO1	IRP1	aconitase 1	ENSG00000122729
ACTA2	αSMA	Actin α 2, Smooth Muscle	ENSG00000107796
AGO1		Argonaute RISC Catalytic Components 1	ENSG00000092847
AGO2		Argonaute RISC Catalytic Components 2	ENSG00000123908
AGO3		Argonaute RISC Catalytic Components 3	ENSG00000126070
AGO4		Argonaute RISC Catalytic Components 4	ENSG00000134698
AKT1	Akt	AKT Serine/Threonine Kinase 1	ENSG00000142208
APC		APC Regulator Of WNT Signaling Pathway	ENSG00000134982
BAX		BCL2 associated X, apoptosis regulator	ENSG00000087088
BCL2	Bcl-2	BCL2 apoptosis regulator	ENSG00000171791
BCL2L1	Bcl-xl	BCL2 Like 1	ENSG00000171552
BECN1		Beclin1	ENSG00000126581
BIRC5	survivin	Baculoviral IAP Repeat Containing 5	ENSG00000089685
BRCA1		BRCA1 DNA Repair Associated	ENSG00000012048
CASC9		Cancer Susceptibility 9	ENSG00000249395
CASP3	caspase-3	Caspase 3	ENSG00000164305
CAV1	CAV-1	Caveolin 1	ENSG00000105974
CCND1		Cyclin D1	ENSG00000110092
CELF1		CUGBP Elav-Like Family Member 1	ENSG00000149187
CD274	PD-L1	CD274 Molecule	ENSG00000120217
CDH1	E-cadherin	Cadherin 1	ENSG00000039068
CDH13		Cadherin 13	ENSG00000140945
CDH2	N-cadherin	Cadherin 2	ENSG00000170558
CDKN1A	p21	CDK Inhibitor 1A	ENSG00000124762
CDKN1B	p27	CDK Inhibitor 1B	ENSG00000111276
CDKN2A	p14, p16, p19	CDK Inhibitor 2A	ENSG00000147889
CDKN2B	p15	CDK Inhibitor 2B	ENSG00000147883
COL1A1		Collagen type I α 1 chain	ENSG00000108821
CBX7		Chromobox 7	ENSG00000100307
CTNNB1	β-catenin	Catenin β 1	ENSG00000168036
CTNND1		Catenin δ 1	ENSG00000198561
CXCL5		C-X-C Motif Chemokine Ligand 5	ENSG00000163735
CXCL8	IL8	C-X-C Motif Chemokine Ligand 8	ENSG00000169429
DACH1		Dachshund Family Transcription Factor 1	ENSG00000276644
DAPK1	DAPK	Death Associated Protein Kinase 1	ENSG00000196730
DGCR8		DGCR8 Microprocessor Complex Subunit	ENSG00000128191
DLK1		δ Like Non-Canonical Notch Ligand 1	ENSG00000185559
DIO3		Iodothyronine Deiodinase 3	ENSG00000197406
DNMT1		DNA Methyltransferase 1	ENSG00000130816
DNMT3B	DNMT3b	DNA Methyltransferase 3 β	ENSG00000088305
DUSP6		Dual Specificity Phosphatase 6	ENSG00000139318
E2F1		E2F Transcription Factor 1	ENSG00000101412
E2F3		E2F Transcription Factor 1	ENSG00000112242
EDNRB		Endothelin Receptor Type B	ENSG00000136160
EED		Embryonic Ectoderm Development	ENSG00000074266
EGFR		Epidermal Growth Factor Receptor	ENSG00000146648
EIF4A3	eIF4AIII	Eukaryotic Translation Initiation Factor 4A3	ENSG00000141543
ELAVL1	HuR	ELAV Like RNA Binding Protein 1	ENSG00000066044
EPAS1		Endothelial PAS Domain Protein 1	ENSG00000116016
ESR2	ERβ	Estrogen Receptor 2	ENSG00000140009
EWSR1		EWS RNA Binding Protein 1	ENSG00000182944
EZH1		Enhancer Of Zeste 1 PRC2 Subunit	ENSG00000108799
EZH2		Enhancer Of Zeste 2 PRC2 Subunit	ENSG00000106462
FAS		Fas Cell Surface Death Receptor	ENSG00000026103
Fgf1		Fibroblast growth factor 1	ENSRNOG00000013867
FMR1	FMRP	FMRP Translational Regulator 1	ENSG00000102081
FXR1		FMR1 Autosomal Homolog 1	ENSG00000114416
FN1		Fibronectin 1	ENSG00000115414
FOXA1	FoxA1	Forkhead Box A1	ENSG00000129514
FOXA2	FoxA2	Forkhead Box A2	ENSG00000125798
FOXF1		Forkhead Box F1	ENSG00000103241
FOXF2		Forkhead Box F2	ENSG00000137273
FOXN1		Forkhead Box N1	ENSG00000109101
FOXP3		Forkhead Box P3	ENSG00000049768
FUS		FUS RNA Binding Protein	ENSG00000089280
HELLS	LSH	Helicase, Lymphoid Specific	ENSG00000119969
HES1		hes family bHLH transcription factor 1	ENSG00000114315
HEY1		hes related family bHLH transcription factor	
		with YRPW motif 1	ENSG00000164683
HIF1A	HIF-1α	Hypoxia Inducible Factor 1 Subunit α	ENSG00000100644
HOXA1		Homeobox A1	ENSG00000105991
HOXA10		Homeobox A10	ENSG00000253293
HOXA11		Homeobox A11	ENSG00000005073
HOXB7		Homeobox B7	ENSG00000260027
HNRNPA2B1	hnRNPA2B1	Heterogeneous Nuclear Ribonucleoprotein A2/B1	ENSG00000122566
HNRNPC		Heterogeneous Nuclear Ribonucleoprotein C	ENSG00000092199
HNRNPL		Heterogeneous Nuclear Ribonucleoprotein L	ENSG00000104824
IGF1R	IGF-IR	Insulin Like Growth Factor 1 Receptor	ENSG00000140443
IGF2		Insulin Like Growth Factor 2	ENSG00000167244
IGF2BP1		IGF2 MRNA Binding Protein 1	ENSG00000159217
IGF2BP2		IGF2 MRNA Binding Protein 2	ENSG00000073792
IGF2BP3		IGF2 MRNA Binding Protein 3	ENSG00000136231
IL1A	IL1α	Interleukin 1 α	ENSG00000115008
IL1B	IL1β	inflammatory cytokines Interleukin 1 β	ENSG00000125538
IL6		Interleukin 6	ENSG00000136244
IL17A	IL17	Interleukin 17A	ENSG00000112115
IL22		Interleukin 22	ENSG00000127318
JARID2		Jumonji And AT-Rich Interaction Domain Containing 2	ENSG00000008083
JUN	c-Jun	Jun Proto-Oncogene, AP-1 Transcription Factor Subunit	ENSG00000177606
KDM1A	LSD1	Lysine Demethylase 1A	ENSG00000004487
KLF2		Kruppel Like Factor 2	ENSG00000127528
KLF4		Kruppel Like Factor 4	ENSG00000136826
KRT14		Keratin 14	ENSG00000186847
KRT19	Cyfra21-1	Keratin 19	ENSG00000171345
LIMK2	LIMK2b	LIM Domain Kinase 2	ENSG00000182541
LIN28B		Lin-28 Homolog B	ENSG00000187772
LRP1		LDL receptor-related protein-1	ENSG00000123384
MACC1		MET Transcriptional Regulator MACC1	ENSG00000183742
MAP1LC3A	LC3I	Microtubule Associated Protein 1 Light Chain 3 α	ENSG00000101460
MAP1LC3B	LC3II	Microtubule Associated Protein 1 Light Chain 3 β	ENSG00000140941
MAPK1	ERK, ERK2, MAPK	Mitogen-Activated Protein Kinase 1	ENSG00000100030
MAPK3	ERK1	Mitogen-Activated Protein Kinase 3	ENSG00000102882
MAPK8	JNK	Mitogen-Activated Protein Kinase 8	ENSG00000107643
MAPK9		Mitogen-Activated Protein Kinase 9	ENSG00000050748
MAP2K1	MEK1	Mitogen-Activated Protein Kinase Kinase 1	ENSG00000169032
MAP2K2	MEK2	Mitogen-Activated Protein Kinase Kinase 2	ENSG00000126934
MCL1		MCL1 Apoptosis Regulator, BCL2 Family Member	ENSG00000143384
MDM2		MDM2 Proto-Oncogene	ENSG00000135679
MDK	MK	Midkine	ENSG00000110492
MIAT		Myocardial Infarction Associated Transcript	ENSG00000225783
MGMT		O-6-Methylguanine-DNA Methyltransferase	MGMT
MMP2	MMP-2	Matrix Metallopeptidase 2	ENSG00000087245
MMP9	MMP-9	Matrix Metallopeptidase 9	ENSG00000100985
MOV10		Mov10 RISC Complex RNA Helicase	ENSG00000155363
MTOR	mTOR	Mechanistic Target Of Rapamycin Kinase	ENSG00000198793
MYC	c-myc	MYC Proto-Oncogene, BHLH Transcription Factor	ENSG00000136997
MYOD1	MYOD	Myogenic differentiation 1	ENSG00000129152
NANOG	Nanog	Nanog Homeobox	ENSG00000111704
NEDD9		Neural Precursor Cell Expressed,	
		Developmentally Down-Regulated 9	ENSG00000111859
NF1		Neurofibromin 1	ENSG00000196712
NFKB1	NF-κB	Nuclear Factor Kappa B Subunit 1	ENSG00000109320
PARP1	PARP	Poly(ADP-Ribose) Polymerase 1	ENSG00000143799
PCNA		Proliferating Cell Nuclear Antigen	ENSG00000132646
PDK1		Pyruvate Dehydrogenase Kinase 1	ENSG00000152256
PDPK1		3-Phosphoinositide Dependent Protein Kinase 1	ENSG00000140992
PHOX2A	Phox2a	Paired Like Homeobox 2A	ENSG00000165462
PIK3CA	PI3K	Phosphatidylinositol-4,5-Bisphosphate	
		3-Kinase Catalytic Subunit α	ENSG00000121879
POU5F1	Oct3, Oct4	POU Class 5 Homeobox 1	ENSG00000204531
PRC1		Polycomb Repressive Complex 1	
PRC2		Polycomb Repressive Complex 2	
PTBP1	PTB	Polypyrimidine Tract Binding Protein 1	ENSG00000011304
PTEN		Phosphatase And Tensin Homolog	ENSG00000171862
PUM2		Pumilio RNA Binding Family Member 2	ENSG00000055917
RB1	Rb	RB Transcriptional Corepressor 1	ENSG00000139687
RELA	p65	RELA Proto-Oncogene, NF-KB Subunit	ENSG00000173039
REST		RE1 Silencing Transcription Factor	ENSG00000084093
RCOR1	CoREST	REST Corepressor 1	ENSG00000089902
RIOX2	mdig	Ribosomal Oxygenase 2	ENSG00000170854
RORA		RAR Related Orphan Receptor A	ENSG00000069667
ROCK2		Rho Associated Coiled-Coil	
		Containing Protein Kinase 2	ENSG00000134318
RORC	RORγt	RAR related orphan receptor C	ENSG00000143365
RPS6KB1	S6K1	Ribosomal Protein S6 Kinase B1	ENSG00000108443
RTL1		Retrotransposon Gag Like 1	ENSG00000254656
SFN	14-3-3σ	Stratifin	ENSG00000175793
SKP2		S-Phase Kinase Associated Protein 2	ENSG00000145604
SLC34A2		Solute Carrier Family 34 Member 2	ENSG00000157765
SMAD3		SMAD family member 3	ENSG00000166949
SNAI1		Snail Family Transcriptional Repressor 1	ENSG00000124216
SNAI2	SLUG	Snail Family Transcriptional Repressor 2	ENSG00000019549
SOX2		SRY-box transcription factor 2	ENSG00000181449
SOX7		SRY-Box Transcription Factor 7	ENSG00000012048
SOX9		SRY-Box Transcription Factor 9	ENSG00000125398
SP1		Sp1 Transcription Factor	ENSG00000185591
SRSF1	SFRS1	Serine And Arginine Rich Splicing Factor 1	ENSG00000136450
STAT3		Signal transducer and activator of transcription 3	ENSG00000168610
SUZ12		SUZ12 Polycomb Repressive Complex 2 Subunit	ENSG00000178691
TARDBP	TDP43	TAR DNA Binding Protein	ENSG00000120948
TBILA		TGF-Beta Induced LncRNA	ENSG00000261488
TCF7		Transcription Factor 7	ENSG00000081059
TFAP2C		Transcription Factor AP-2 γ	ENSG00000087510
TGFB1	TGFβ1	transforming growth factor β 1	ENSG00000105329
TIA1		TIA1 Cytotoxic Granule Associated RNA Binding Protein	ENSG00000116001
TIAL1		TIA1 Cytotoxic Granule Associated RNA Binding Protein Like 1	ENSG00000151923
TIMP2		TIMP Metallopeptidase Inhibitor 2	ENSG00000035862
TNF	TNFα	Tumor Necrosis Factor	ENSG00000232810
TNRC6A	TNRC6	Trinucleotide Repeat Containing Adaptor 6A	ENSG00000090905
TP53		tumor protein p53	ENSG00000141510
TP63		tumor protein p63	ENSG00000073282
TP73		tumor protein p73	ENSG00000078900
U2AF2	U2AF65	U2 Small Nuclear RNA Auxiliary Factor 2	ENSG00000063244
ULK1		Unc-51 Like Autophagy Activating Kinase 1	ENSG00000177169
UPF1		UPF1 RNA Helicase And ATPase	ENSG00000005007
VIM		vimentin	ENSG00000026025
WNT10A	Wnt 10a	Wnt Family Member 10A	ENSG00000135925
WNT2	Wnt 2	Wnt Family Member 2	ENSG00000105989
WNT3A		Wnt Family Member 3A	ENSG00000154342
WNT5A	Wnt 5a	Wnt Family Member 5A	ENSG00000114251
WNT6	Wnt 6	Wnt Family Member 6	ENSG00000115596
YAP1		Yes associated protein 1	ENSG00000137693
ZC3H7B		Zinc Finger CCCH-Type Containing 7B	ENSG00000100403
ZEB1		Zinc Finger E-Box Binding Homeobox 1	ENSG00000148516
ZEB2		Zinc Finger E-Box Binding Homeobox 2	ENSG00000169554
